# Molecular and Electrophysiological Characterization of a Novel Cation Channel of *Trypanosoma cruzi*

**DOI:** 10.1371/journal.ppat.1002750

**Published:** 2012-06-07

**Authors:** Veronica Jimenez, Roberto Docampo

**Affiliations:** Center for Tropical and Emerging Global Diseases and Department of Cellular Biology, University of Georgia, Athens, Georgia, United States of America; University of Michigan, United States of America

## Abstract

We report the identification, functional expression, purification, reconstitution and electrophysiological characterization of a novel cation channel (TcCat) from *Trypanosoma cruzi*, the etiologic agent of Chagas disease. This channel is potassium permeable and shows inward rectification in the presence of magnesium. Western blot analyses with specific antibodies indicated that the protein is expressed in the three main life cycle stages of the parasite. Surprisingly, the parasites have the unprecedented ability to rapidly change the localization of the channel when they are exposed to different environmental stresses. TcCat rapidly translocates to the tip of the flagellum when trypomastigotes are submitted to acidic pH, to the plasma membrane when epimastigotes are submitted to hyperosmotic stress, and to the cell surface when amastigotes are released to the extracellular medium. Pharmacological block of TcCat activity also resulted in alterations in the trypomastigotes ability to respond to hyperosmotic stress. We also demonstrate the feasibility of purifying and reconstituting a functional ion channel from *T. cruzi* after recombinant expression in bacteria. The peculiar characteristics of TcCat could be important for the development of specific inhibitors with therapeutic potential against trypanosomes.

## Author Summary

The use of high-resolution electrophysiological techniques to study ion channels has provided a large amount of information on functional aspects of these important membrane proteins. However, the study of ion channels in unicellular eukaryotes has been limited to detection of ion conductances in large cells, gene identification studies, and pharmacological treatments to investigate the potential presence of different ion channels. In this paper we report the first identification, functional expression, purification, reconstitution, and electrophysiological characterization with single-molecule resolution of a novel cation channel (TcCat) from *Trypanosoma cruzi*. This is a novel channel that shares little sequence and functional similarities to other potassium channels and its peculiar characteristics could be important for the development of specific inhibitors with therapeutic potential against trypanosomiasis. Surprisingly, the parasites have the unprecedented ability to rapidly change the localization of the channel when they are exposed to different environmental stresses. We demonstrated the feasibility of purifying and reconstituting a functional ion channel from *T. cruzi* after recombinant expression in bacteria. In addition, we obtained yeast mutants that will provide a useful genetic system for studies of the assembly and composition of the channel.

## Introduction

*Trypanosoma cruzi* is a unicellular parasitic eukaryote and the etiologic agent of Chagas disease, which currently affects millions of people in North, Central and South America, and is becoming frequently diagnosed in non-endemic countries [1], [2].

*T. cruzi* has a complex life cycle involving insect and mammalian hosts and different morphological and functional stages: epimastigotes and metacyclictrypomastigotes in the insect vector, and intracellular amastigotes and bloodstream trypomastigotes in the mammalian host. During its life cycle, the parasite finds extreme fluctuations in environmental conditions to which it must adapt in order to survive. A wide range of ionic concentrations, osmolarities and pHs are major challenges to cope with when it transits through the vector gut to the excreta, and from this highly concentrated environment to the interstitial fluid of the mammalian host. Particularly, the concentration of K^+^in the vector can vary between 40 to 358 mM depending on the feeding cycles of the insect [3], and from 5 to 140 mM between the extra and intracellular environments of the mammalian stages.

In previous studies [4], [5] we demonstrated that a plasma membrane H^+^-ATPase is the major regulator of intracellular pH (pH_i_) in all stages of *T. cruzi*. However, in contrast to epimastigotes, whose pH_i_ is not affected by extracellular cations [4], trypomastigotes possess a cation-dependent pH_i_ control. We proposed [5] that, as occurs in plants [6], [7] and other protists [8], in these trypomastigote stages an inward rectifier K^+^ channel functions in K^+^ uptake dissipating the plasma membrane potential(*Vm*) generated by the H^+^-ATPase thereby increasing its efficiency. This putative channel could be blocked bythe addition of Cs^2+^ or Ba^2+^ [5].The plasma membrane H^+^-ATPase also plays a significant role in the regulation of *Vm* in all stages of *T. cruzi* [9]. In contrast to epimastigotes the *Vm* of trypomastigotes is markedly sensitive to extracellular Na^+^ and K^+^. In support of the presence of a K^+^permeable channel, the *Vm* is hyperpolarized by K^+^-free buffer in trypomastigotes [9]. Interestingly, trypomastigotes are able to maintain a negative *Vm* in a K^+^-rich buffer at acidic pH, conditions that they encounter when they enter the parasitophorous vacuole [9]. This is differentfrommammalian cells, which are usually depolarized by either acidic or high extracellular K^+^ concentrations. Amastigotes, in contrast, appear to be impermeable to K^+^ in agreement with the high intracellular K^+^ environment in which they live [9]. The marked differences in the regulation of *V_μ_* in trypomastigotes as compared to amastigotes suggest that during transformation to amastigotes, trypomastigotes undergo significant changes in their ion transport mechanisms. However, the nature of these changes and the molecular identity of K^+^permeable pathways are unknown.

K^+^ channels are members of one of the largest and most diverse families of membrane proteins, widely described from bacteria to humans [10]–[12]. Their roles include plasma membrane potential maintenance, pH_i_ and cell volume regulation, excitability, organogenesis and cell death [13]–[16]. From the structural point of view, they can be divided into two main groups: channels containing six transmembrane domains, including in this category voltage-dependent K^+^ channels and calcium-activated K^+^ channels [17], and channels with only two transmembrane domains, such as the inward rectifier K^+^channels (Kir channels) [18] and the widely described bacterial channel KcsA [10]. As a general rule, a functional K^+^ channel is formed by interaction of four pore-forming subunits interacting through a conserved tetramerization domain. Association with other proteins, interaction with surrounding lipids and post-translational modifications generate a functional diversity that exceeds the predictions based solely on the number of identified genes [17].

High yield recombinant expression and purification of functional ion channels has been technically very difficult and restricted to prokaryotic channels until recently [19]. In this work we demonstrate the feasibility of purifying a functional cation channel from *T. cruzi* after recombinant expression in bacteria. We report the molecular and electrophysiological characteristics of this inwardly rectifying K^+^permeable channel and the changes occurring in its localization during the parasitetransformation into different developmental stages. Our results indicate that *T. cruzi* has the unexpected ability to change the localization of this cation channel to adapt to different environments to which it is exposed in its different developmental stages.

## Results

### Cloning and sequencing of *TcCat*

We searched for K^+^channels in the TriTryp database (http://tritrypdb.org/tritrypdb/) and found two genes encoding for putative voltage-dependent K^+^channels in *T. cruzi* (Tc00.1047053511301.140 and Tc00.1047053507213.30). The sequences showed 98% identity between them and likely correspond to alleles of the same gene (*TcCat*). The orthologous identified in *T. brucei* (Tb927.10.16170) and *L. major* (LmjF19.1620) shared 64% and 55% amino acid identity respectively (Fig. S1A). Structural analysis (TopPred) (Text S1)predicted two transmembrane domains between amino acids 77–97 and 169–189 and a tetramerization domain at position 5–73 (Pfam02214) that is the only region with similarity to other K^+^channels like Kv4.3 (Fig. S1B). The ORF predicts a 297 amino acid protein with an apparent molecular weight of 34 kDa. No significant identity was found with well-characterized bacterial channels like KcsA or with mammalian (Kir channels)and bacterial inward-rectifiers (Fig. S1C). Interestingly, no conserved K^+^ channel signature sequence [T-X-G-Y(F)-G] [20] was identified in TcCat, raising the question of the ion selectivity of this channel. Other important features of TcCat are the presence of longer mode 2 interacting phospho-motif for 14-3-3 proteins at positions 128–134 (RHALTIT), putative phosphorylation sites at serines 103, 190, 214 and 248 and *N*-glycosylation sites at positions 181 (NGTA), 228 (NFTF) and 286 (NSTR), that can be relevant for the regulation of the activity and the interaction with other proteins.

### TcCat localization in different life stages

**Figure 1:**
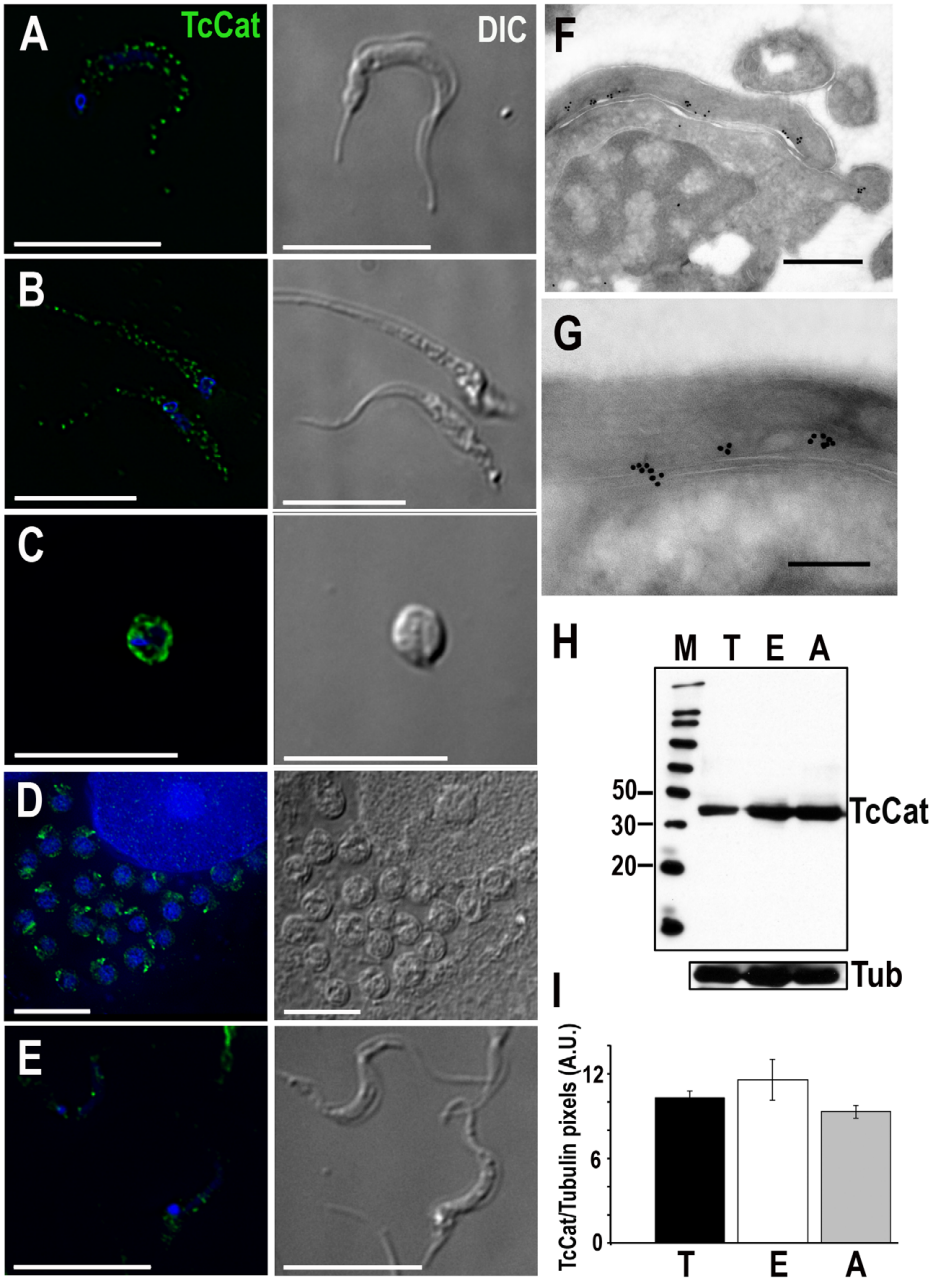
TcCat expression and localization in *T. cruzi* life stages. TcCat immunolocalization (*green*) in *T. cruzi* trypomastigotes (**A**), epimastigotes (**B**), extracellular amastigotes (**C**), intracellular amastigotes (**D**) and metacyclictrypomastigotes (**E**). Nuclei were DAPI stained. Bars = 10 µm. **F, G**. Immunoelectron microscopy localization of TcCat in trypomastigotes with purified anti-TcCat and secondary anti-rabbit gold-labeled antibody. Bars: **F** = 0.5 µm, **G** = 0.2 µm. **H**. Western blot analysis of TcCat expression in *T. cruzi* homogenates. Lanes: M: molecular weight markers in kDa (MagicMark XP, Invitrogen), T: trypomastigotes, E: epimastigotes, A: extracellular amastigotes. Bottom: membranes were stripped and re-incubated with anti-tubulin antibody as a loading control. **I**. Densitometry of TcCat detection by western blot analysis in arbitrary units (AU). T: trypomastigotes, E: epimastigotes, A: extracellular amastigotes. Values in arbitrary units (AU) correspond to mean ± SEM from 3 independent experiments.

TcCat localization was analysed by indirect immunofluorescence using affinity-purified antibodies against the recombinant protein. In trypomastigotes, the channel has a clearly defined punctuate pattern along the flagellum (Fig. 1A). In epimastigotes (Fig. 1B), TcCat also has a peripheral punctuated pattern with some apparently intracellular labeling. To further evaluate whether the punctuate localization could be due to labeling of patches of plasma membrane and not intracellular vesicles we performed immunolocalization in permeabilized and non-permeabilized cells. In both trypomastigotes and epimastigotes TcCat was detected, at least in part, exposed to the cell surface (Fig. S2). In amastigotes that were spontaneously released to the supernatant of infected L_6_E_9_ myoblasts the channel showed plasma membrane localization (Fig. 1C). However, in intracellular amastigotes TcKCat seems to be confined to a spot that could be the remaining short flagellum (Fig. 1D). This change in localization is consistent with a role of TcCat in K^+^ uptake, which would become less important in the intracellular environment rich in K^+^. In agreement with a developmental regulation of TcCat expression, labeling decreased considerably in metacyclictrypomastigotes (Fig. 1E). Immunoelectron microscopy analysis confirmed the patched distribution of TcCat in tissue culture-derived trypomastigotes along the flagellar attachment zone (Figs. 1F and 1G). The association of ion channels in clusters has been described previously [21]–[23] and seems to be related with preferential targeting to specific membrane lipid microdomains or lipid rafts, which are known to be more abundant in the flagellar membrane of trypanosomes [24].

Expression of TcCat was verified by western blot analysis (Fig. 1H) confirming the presence of the channel in all three stages of the parasite. The native protein detected in the parasites has an apparent molecular mass of 43 kDa, slightly higher than that predicted by the ORF. This difference could be due to post-translational modifications as can be expected from the presence of several putative phosphorylation and N-glycosylation sites. Densitometry analysis using α-tubulin as a loading control as well as Coomassie blue staining (Fig. S3) indicated that the level of expression is similar in trypomastigotes, amastigotes and epimastigotes (Fig. 1I), as suggested by IFA.

**Figure 2:**
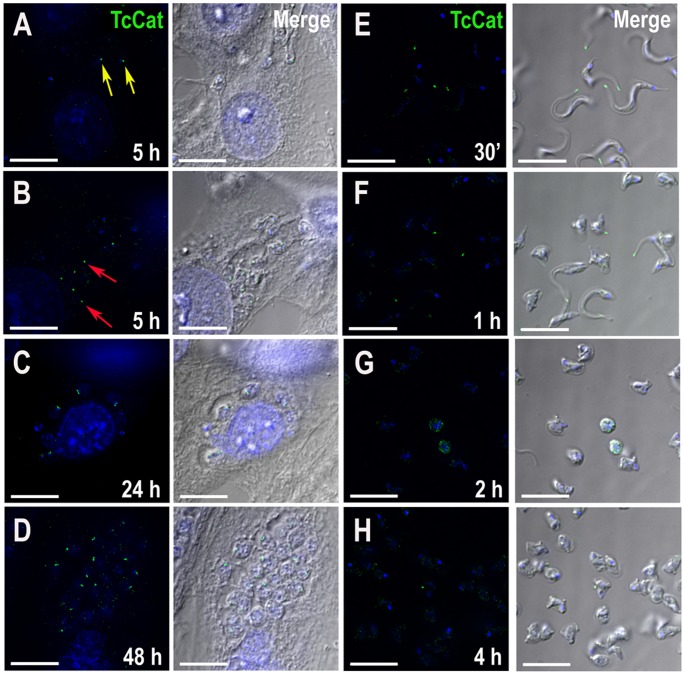
Changes in TcCat localization during differentiation. TcCat immunolocalization (*green*) at different time points after mammalian cell infection (**A–D**) or during *in vitro* differentiation of trypomastigotes to amastigotes at acidic pH (**E–H**). *Yellow arrows* indicate trypomastigote-like morphology and *red arrows* indicate amastigote-like forms at 5 h post-infection. Nuclei were DAPI stained (*blue*). Bars = 10 µm.

We evaluated the change in the localization of TcCat by IFA during the differentiation *in vivo*. At 5 h post-infection of mammalian cells, TcCat is already detected at a single intracellular spot both in parasites with trypomastigote-like morphology (Fig. 2A, *yellow arrows*) and in rounded amastigote-like cells (Fig. 2B, *red arrows*). At 24 and 48 h post-infection (Figs. 2C and 2D) TcCat remains intracellular in the replicating amastigotes, close to the flagellar pocket. In extracellular trypomastigotes, 96 h post-infection, TcCat was always localized at the plasma membrane (Fig. 1A).

We studied the expression of TcCat during the differentiation of trypomastigotes to amastigotes *in vitro*. At 30 min after induction of differentiation *in vitro* at pH 5.0, staining with antibodies against TcCat was at the tip of the flagellum (Fig. 2E), and this single spot labeling was maintained even when the cells rounded up to transform into amastigotes (Figs. 2F–H).

### *TcCat* complements K^+^ influx defective yeast mutants

**Figure 3:**
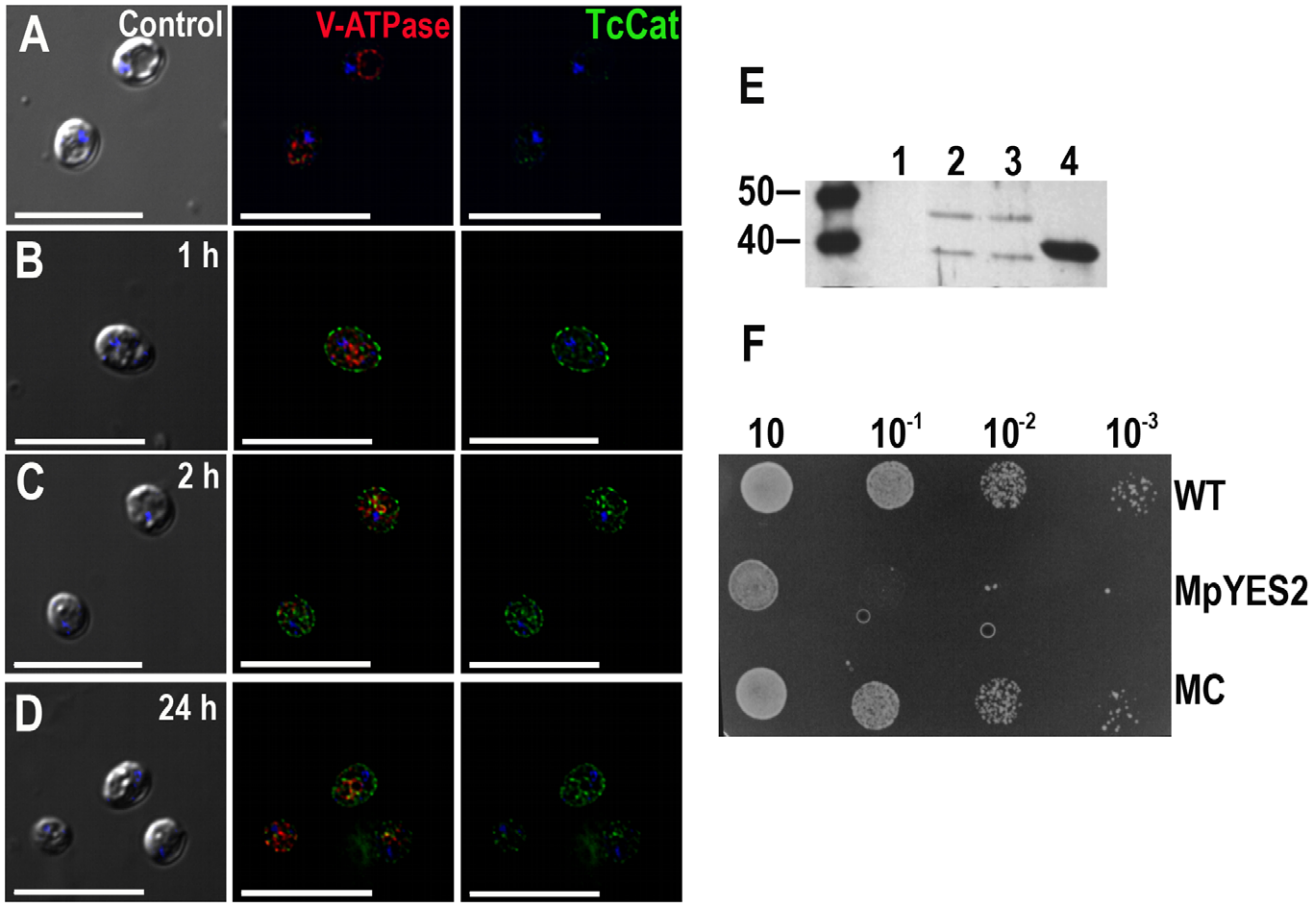
Functional yeast complementation with TcCat. **A–D**. TcCat expression, as analyzed by immunofluorescence, at different times after induction. Yeast were collected at the indicated times and incubated with anti-TcCat antibodies (*green*) and anti-vacuolar H^+^ ATPase (*red*) as a control for proper permeabilization. Nuclei were DAPI stained (*blue*). *Left* panels are DIC images, *right* panels are anti-TcCat stained cells and *central* panels are merge immunofluorescence images. Bars = 10 µm. **E**. Western blot analysis of yeast homogenate with specific anti-TcCat antibody. Lanes, 1: control non-complemented mutant yeast, 2: wild-type strain complemented with TcCat, 3: mutant strain complemented with TcCat, 4: TcCat recombinant protein. **F**. Growth-assay of complemented yeast in SC ura- galactose agar plates. Serial dilutions of initial cultures at OD_600_ = 0.6 were incubated for 72 h at 30°C. WT corresponds to wild type strain, MpYES2 is the mutant transformed with the empty vector pYES2, MC represents the mutant strain transformed with TcCat-pYES2 construct.

Potassium uptake defective *S. cerevisiae* mutants (*Δtrk1, Δtrk2, Δtok1*) were used to investigate the K^+^ influx ability of TcCat (see Text S1). These mutants depend on high extracellular K^+^ concentration for their growth as they only have the non-specific cation uptake mechanism, termed NSC1, for growth [25]. Mutants were kept in defined medium (SC ura-) supplemented with 100 mMKCl, pH 5.8. TcCat expression was induced by switching the carbon source and the channel was rapidly detected on the yeast surface by immunofluorescence analysis with anti-TcCat antibodies (Fig. 3B). After 2 h (Fig. 3C), yeasts were collected by centrifugation and placed in standard SC ura-medium without KCl. Otherwise the presence of high K^+^ concentration was toxic upon induction of *TcCat* expression. The channel was expressed on the yeast surface for up to 72 h at high levels, although, at 24 h, some labeling could be observed in the periphery of the yeast vacuole, probably due to recycling or degradation (Fig. 3D). Control cells were transformed with empty vector pYES2. A monoclonal antibody against the 69-kDa subunit of the vacuolar H^+^-ATPase was used as a control of proper permeabilization (Fig. 3A–D).

*TcCat* expression in complemented yeasts was also verified by western blot analysis using anti-TcCat antibodies (Fig. 3E). Two bands were detected, one that corresponds to the predicted molecular weight of the protein product (about 35 kDa) and a second band of approximately 45 kDa similar to that of the native protein in the parasites (Fig. 1H), suggesting that post-translational modifications also occurred in yeasts.

Functional complementation and restoration of the normal growth phenotype was achieved when culturing the mutant yeast in serial dilutions in SC ura-galactose agar plates without addition of KCl. Under these conditions, mutants transfected with vector alone (MpYES2) were not able to grow when diluted to 10^−1^ or more, while mutants complemented with *TcCat*(MC) showed no significant difference in growth as compared with wild type yeast (WT) (Fig. 3F). The results indicate that TcCat is indeed a K^+^ conductive pathway able to functionally complement a heterologous system.

### TcCat electrophysiological characterization

**Figure 4:**
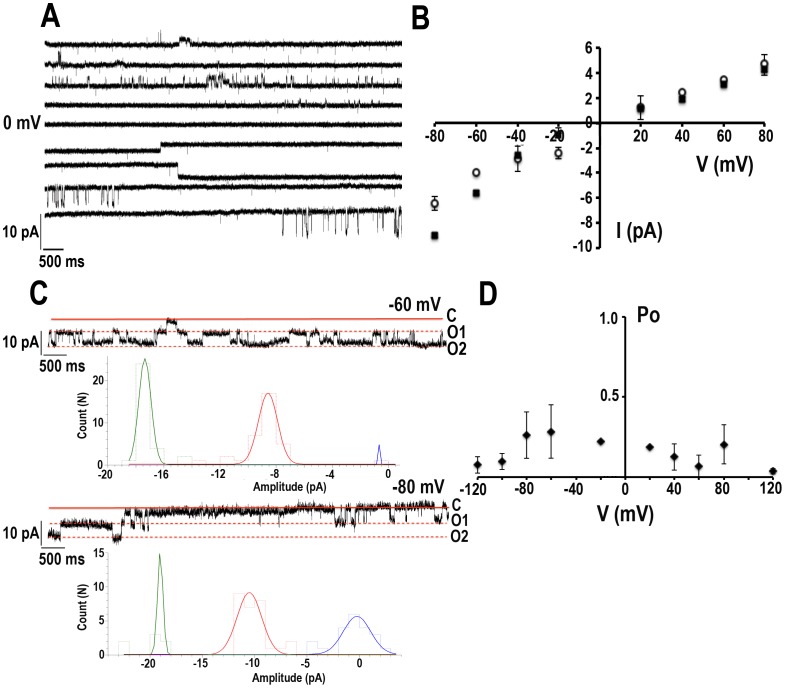
Biophysical characterization defines TcCat as an inward rectifier channel. **A**. Representative current traces applying a voltage-step protocol from −80 to 80 mV in 20 mV steps. The recordings were obtained under symmetrical conditions in the absence of Mg^2+^ with bath and pipette solution containing 140 mMKCl, 10 mMHepes-K, pH 7.4. **B**. Current-voltage relationship under symmetrical conditions described in (**A**). Data correspond to the unitary currentsrecorded in continuous voltage steps at the indicated holding potentials in the absence (*open circles*, n = 14) or in the presence of 1 mM MgCl_2_ in the bath solution (*solid squares*, n = 13). The non-linear relationship indicates the inward rectification. **C**. Current traces obtained at the indicated holding potentials showing the functional association of TcCat in clusters under asymmetrical conditions with bath solution 300 mMKCl and pipette solution 140 mMKCl, both containing 10 mMHepes-K, pH 7.4. *Doted lines* indicate the open state of the two channels (O_1_ and O_2_) present in the seal. C indicates the closed state of the channels. Histograms represent the unitary current corresponding to one or 2 channels at the indicated voltages. **D**. Open probability analysis of the single channel events. Values correspond to mean ± SEM from 14 independent experiments.

The activity of TcCat was detected in patches excised from cell-size giant liposomes (inside-out configuration) containing the purified recombinant protein (Text S1, Figs. S4 and S5, and Table S1).Currents from liposomes containing only asolectin were recorded as control (Fig. S6A,*black squares*), showing a significant lower level compared with currents from liposomes containing purified TcCat (Fig. S6A, *red circles*). Currents were recorded under symmetrical conditions in the absence of Mg^2+^, unless stated otherwise, with bath and pipette solutions containing 140 mMKCl, 10 mMHepes-K, pH 7.4. Single channel currents were observed when an increasing voltage-pulse protocol between −80 to +80 mV was applied (Fig. 4A). The current-potential relationship for the single channel was not linear in the presence of Mg^2+^, as expected for an inward rectifier channel. The chord conductance (γ) (Fig. 4B, *open circles*) calculated under symmetrical KCl in the absence of Mg^2+^was 77±4 pS and 59±2 pS at −80 and +80 mV, respectively (n = 14) indicating a slight intrinsic rectification. Although no significant reduction in the current was observed at positive potentials in the presence of Mg^2+^, a significant increase in the inward current was evident at negative potentials in the presence of 1 mM MgCl_2_ in the bath solution (Fig. 4B, black squares), with unitary conductances of 122±7 pS and 56±3 pS at −80 and +80 mV, respectively (n = 13). These results suggest that the mechanism of blockage by Mg^2+^ is different from the one described for inward rectifier K^+^ channels. The unitary level of current was frequently observed in clusters, as shown in Fig. 4C where at least two channels could be detected, opening and closing independently. The histograms showncorrespond to the unitary current of one or two channels at the indicated voltages (Fig. 4C). This recorded activity agrees well with the localization in patches described above. Important variations in the open probability were observed in recordings from different days. When 14 independent experiments were analyzed, the open probability was not significantly sensitive to voltage, with values of 0.26±0.04 and 0.2±0.04 at −80 and +80 mV, respectively (Fig. 4D).

**Figure 5:**
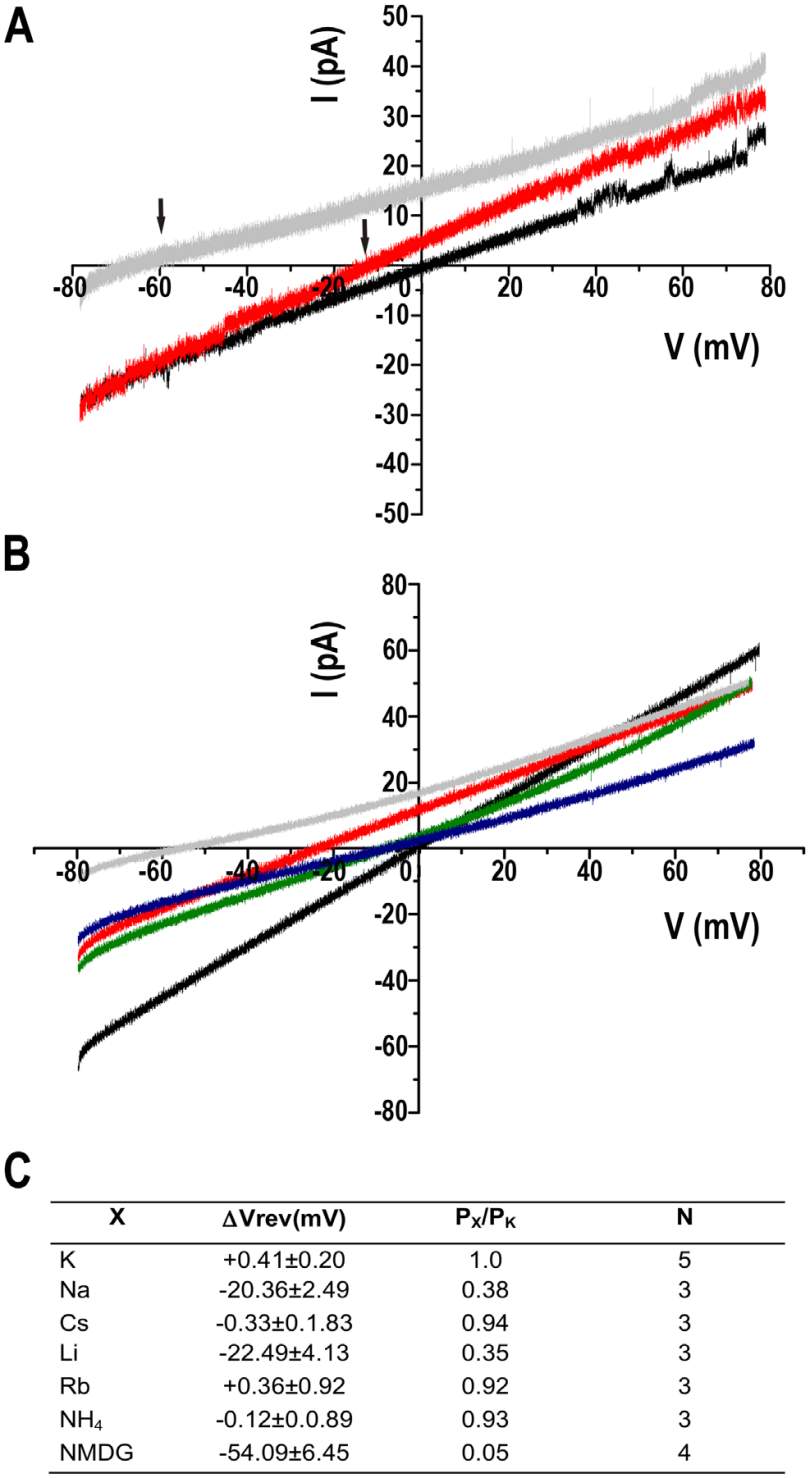
Ion selectivity of TcCat. **A**. Representative current traces recorded under a voltage-ramp protocol between −80 and +80 mV. *Back line* corresponds to symmetrical conditions in the absence of Mg^2+^ (bath and pipette solution 140 mMKCl, 10 mMHepes-K, pH 7.4). *Grey line* represents the current under non-symmetrical conditions, replacing the bath solution for 140 mM NMDG-Cl, 10 mMHepes-K, pH 7.4. *Red line* shows the current trace when the bath solution contains 140 mM K-gluconate, 10 mMHepes-K, pH 7.4. *Arrows* indicate the shift in the reversal potential of the current for asymmetrical conditions. **B**. Current traces recorded applying a voltage-ramp protocol between −80 to +80 mV under symmetrical conditions described in (**A**) (*black line*) or replacing the bath solution for 140 mMXCl, 10 mMHepes-K, pH 7.4, X being Na^+^ (*red line*), Cs^+^ (*green line*), NH_4_^+^ (*blue line*) or NMDG (*grey line*). **C**. Relative permeability ratios for monovalent cations (X) respect to K^+^ (P_X_/P_K_). *ΔVrev* corresponds to the difference between the reversal potential of the current for the control and the experimental shift in reversal potential when replacing the monovalent cation in the bath solution. Values are expressed as mean ± SEM. N indicates independent experiment. Each experimental value is the average of 5 measurements for each experiment.

The cationic nature of the TcCat conductive properties was verified applying a voltage-ramp protocol from −80 to +80 mV under symmetrical conditions(Fig. 5A, *black line*) or replacing the bath solution for a non-permeantcation (140 mM NMDG-Cl, 10 mMHepes-K, pH 7.4 (Fig. 5A, *gray line*). A shift in the reversal potential of the current (*ΔV_rev_*) was observed from 0 mV to −54±6 mV (n = 4), close to the theoretical *V_rev_* calculated for K^+^ under those conditions (−70 mV). Replacement of the bath solution for buffered 340 mMKCl induced a *ΔV_rev_* of +39±1 mV (n = 5) with a theoretical calculated *V_rev_* of +22 mV. These results suggest that TcCat preferentially permeates K^+^. To calculate the selectivity for cations over anions of TcCat, we applied similar voltage ramp protocols but replacing the bath solution for a non-permeant anion (140 mM K-gluconate, 10 mMHepes-K, pH 7.4). A *ΔV_rev_* of −8.7±0.4 (n = 10) was measured under asymmetrical conditions (Fig. 5A, *red line*). Based on the bi-ionic equation (see Equations under Text S1), the calculated permeability ratio for K^+^ over Cl^−^ was 5.9±0.5 (n = 10), indicating a preferential cation permeability but with weak selectivity filter, in agreement with the sequence data.

In order to study TcCat selectivity for monovalent cations, a voltage-ramp protocol was applied under symmetrical condition for K^+^ (Fig. 5B, *black line*) or replacing the bath solution for 140 mMXCl, X being different cations. Under bi-ionic conditions, the shift in the *V_rev_* indicates the relative permeability of X^+^ respect to K^+^ (Fig. 5B). The permeability sequence obtained was: K^+^>Cs^+^>NH_4_^+^>Rb^+^>Na^+^>Li^+^>NMDG^+^ (Fig. 5C) that corresponds to Eisenman sequence IIIa [26]. This represents a selectivity of about 2.5 for K^+^ over Na^+^, which may indicate that TcCat is a potential conductive pathway for both physiological ions.

This biophysical characterization shows that TcCat is, indeed, a channel permeable to K^+^ and that shows inward rectification. In the presence of Mg^2+^, the unitary conductance is, as expected, higher at negative than at positive potentials and the open probability is not voltage-dependent.

### Blockage effects

**Figure 6:**
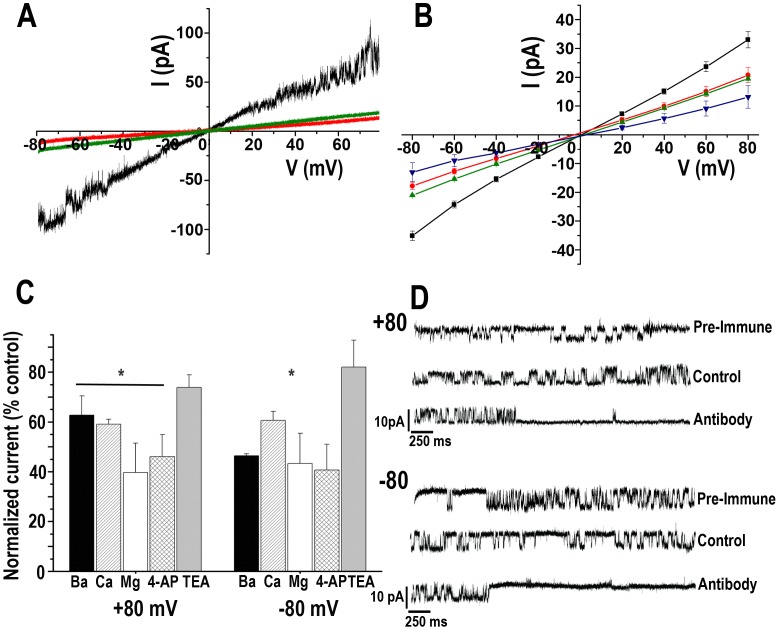
Effect of different blockers on TcCat currents. **A**. Representative current traces recorded under a voltage-ramp protocol between −80 and + 80 mV, under control symmetrical conditions (*black line*) or in the presence of 10 mM BaCl_2_ (*red line*) or CaCl_2_ (*green line*). After applying BaCl_2_, the seal was washed and once the current was back to the levels recorded under control conditions, CaCl_2_ was applied. **B**. Voltage-current relationship obtained from total currents of the seal recorded applying a voltage-step protocol from −80 to +80 mV under symmetrical control conditions for K^+^ (*black squares*), or in the presence of 1 mM BaCl_2_ (*red circles*), 1 mM CaCl_2_ (*green triangles*) or 1 mM MgCl_2_. **C**. Normalized total currents respect to the total current of the seal recorded at the indicated holding potentials in the presence of blockers: 1 mM BaCl_2_, 1 mM CaCl_2_, 1 mM MgCl_2_, 1 µM 4-AP, 10 mM TEA. A significant reduction in the current was found in the presence of the indicated blockers (p<0.001, n = 3 independent experiments). **D**. Current traces recorded applying a pulse protocol at the indicated holding potentials in control conditions (140 mMKCl, 10 mMHepes-K, pH 7.4), in the presence of pre-immune serum, or in the presence of specific anti-TcCat antibody in the bath solution at a concentration of 0.12 mg/ml.

The effect of divalent cations was evaluated by adding controlled concentrations of Ba^2+^, Ca^2+^or Mg^2+^to the bath solution. Fig. 6A shows that in the presence of 10 mM BaCl_2_ (*red line*) or 10 mM CaCl_2_ (*green line*) a significant decrease in the total current can be observed compared with the control (*black line*). No important shift in the *V_rev_* was recorded, indicating the low permeability for these ions. A consistent decrease in the total current was observed when a voltage-step protocol was applied (Fig. 6B) in the presence of lower concentrations of the divalent cations, with a more remarkable effect for Mg^2+^ (*blue inverted triangles*). A concentration-dependent effect was observed for Ba^2+^ and Ca^2+^ when applying a voltage-step protocol in the presence of controlled concentrations of both ions (Fig. S6 C and D). The effect of Ba^2+^ over the leak through asolectin vesicles was evaluated comparing the normalized current in the presence of different concentrations of the divalent ion on empty liposomes (Fig. S6B,*black circles*) or liposomes containing TcCat (Fig. S6B,*black squares*). In the presence of 1 mM BaCl_2_ the residual current at −80 mV (Fig. S6B,*upper panel*) is about 55% in empty liposomes while it is close to 30% in liposomes containing TcCat, indicating that a percentage of the current through the channel is sensitive to the presence of the divalent cation. Similar results are obtained at +80 mV (Fig. S6B,*lower panel*).Based on the dose-dependent blockage, the calculated inhibition constants (K_i_) for Ba^2+^ were (in mM): 0.54±0.08 and 0.63±0.06 at −80 and +80 mV, respectively, with no significant dependency on the applied voltage (n = 3 independent experiments).

The blockage by Ca^2+^ required higher concentration, with calculated *K_is_* (in mM) of 5.2±0.3 and 4.5±0.2 at −80 and +80 mV, respectively. In all cases, a residual current was observed, even at 10 mM divalent cation concentration, indicating a leaking current or a partial blockage (Fig. S6D and F).

Conventional K^+^ channel blockers were also tested (Fig. 6C), with no significant effect for tetraethylammmonium (TEA) up to 10 mM (n = 3) and a 50% reduction in the total current for 4-aminopyridine (4-AP) at 1 µM (n = 4). Importantly, a blockage effect was observed in the presence of the anti-TcCat antibody when added to the bath solution at a concentration of 0.12 µg/µl and at holding potentials of −80 and +80 mV (Fig. 6D). No significant effect was observed when the same concentration of pre-immune sera was applied to the preparations (Fig. 6D, *pre-immune*).

### Role of TcCat in osmotic stress responses

**Figure 7:**
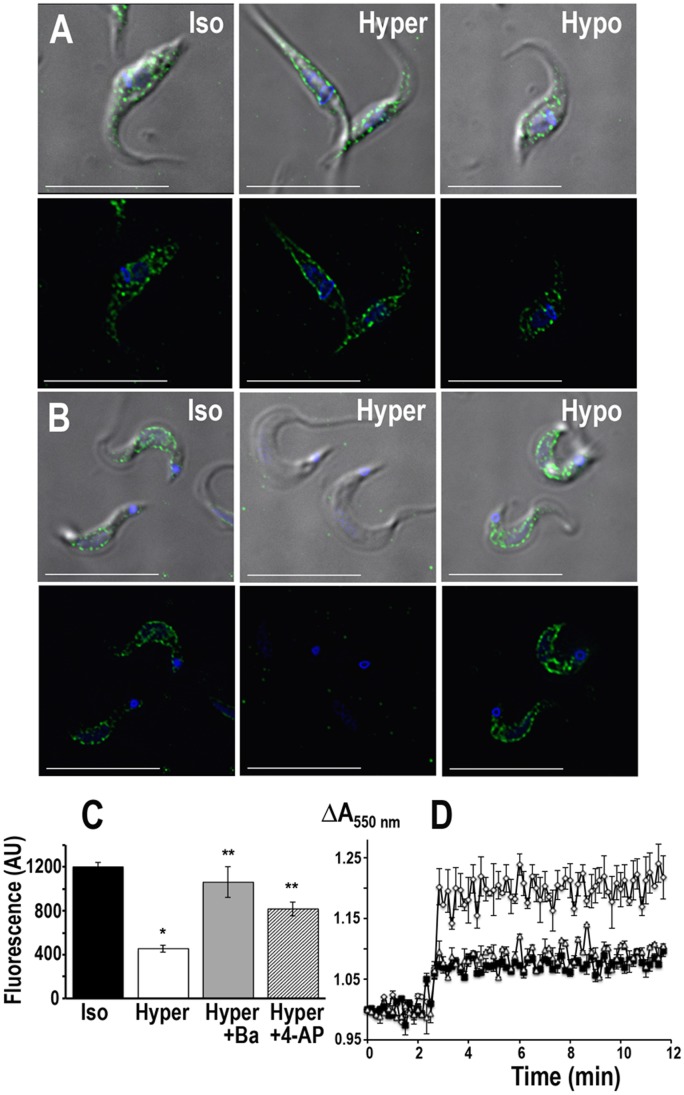
Osmotic stress effect of TcCat localization. TcCat immunolocalization in *T. cruzi* epimastigotes (**A**) and trypomastigotes (**B**) under isosmotic (Iso), hyperosmotic (*Hyper*) or hyposmotic (*Hypo*) conditions. TcCat was detected with purified specific antibody and secondary anti-rabbit Alexa-488 conjugated (*green*). DNA was stained with DAPI (*blue*). Bars = 10 µm. **C**. Quantification of the TcCat label intensity in trypomastigotes under isosmotic or hyperosmotic conditions. Values are expressed in arbitrary units (AU) as mean ± SEM of n = 3 independent experiments. For each experiment and treatment, the pixel intensity of 75 parasites was measured. *P<0.01 respect to the isosmotic condition. **P<0.01 respect to hyperosmotic in the absence of the blockers. **D**. Relative change in trypomastigotes cell volume under hyperosmotic stress (control, *open diamonds*). TcCat blockers significantly reduce the shrinkage (1 mM BaCl_2_ *open triangles*; 100 µM 4-AP, *black squares*). Values are mean ± SEM of n = 3 independent experiments.

As mentioned before, an inward-rectifier K^+^ channel seems to be involved in the maintenance of plasma membrane potential, intracellular pH and osmoregulation [5], [9]. To assess the role of TcCat on some of these processes we evaluated the localization of the protein in *T. cruzi* epimastigotes and trypomastigotes under osmotic stress. Under isosmotic conditions, the channel is localized at the plasma membrane, in a punctuate pattern, with some intracellular staining more evident in epimastigotes (Fig. 7A and B, *Iso*). When epimastigotes were placed under hyperosmotic stress, maintaining the same ionic concentrations, TcCat almost completely translocated to the plasma membrane (Fig. 7A, *Hyper*). Remarkably, in trypomastigotes, after 30 sec under hyperosmotic stress, TcCat disappeared from the cell surface of the parasites (Fig. 7B, *Hyper*). No intracellular accumulation of the protein was observed suggesting that the protein is released to the extracellular medium, probably by shedding mechanisms previously described for other *T. cruzi* surface proteins [27]. To prove this hypothesis, the supernatant of parasites under different osmotic conditions were precipitated and evaluated by western blot analysis. In trypomastigotes under hyperosmotic stress TcCat was detected in the supernatants (Fig. S7A). That was not the case for trypomastigotes under isosmotic or hyposmotic conditions (Fig. S7A) or for epimastigotes under similar treatments (Fig. S7B). Parasites overexpressing GFP were used as a control to rule out lysis of the parasites as a mechanism of release of TcCat(Fig. S7A).

Addition of Ba^2+^ or 4-AP, at concentrations that inhibit TcCat activity, could prevent the change in the localization of the protein in trypomastigotes(Fig. 7C) suggesting that the mechanism of elimination is linked to the sensing of the K^+^ concentration.

No differences were observed in TcCat localization when the parasites were under hyposmotic stress (Fig. 7A and B, *Hypo*). Morphologically, the typical response toosmotic stress can be detected in the parasites, with a more accentuated change in the shape of epimastigotes compared with trypomastigotes (Fig. 7A and 7B). Although the osmotic stress response is sensitive to tubulin de-polymerizing agents [28], TcCat translocation was not blocked or modified by treatment with trifluralin (500 µM) or chloralin (10 µM) (Fig. S8).

The potential role of TcCat in osmoregulation was further evaluated in the parasites under osmotic stress. Interestingly, the shrinkage of trypomastigotes under hyperosmotic stress was significantly reduced in the presence of 1 mM BaCl_2_ (Fig. 7D, *open triangles*) or 100 µM 4-AP (Fig. 7D, *black squares*) as compared to the control (Fig. 7D, *open diamonds*) suggesting a role of K^+^ influx through TcCat during cell volume decrease.

## Discussion

In *T. cruzi* [9] as well as in *T. brucei* [29]–[31] and other protists like *Toxoplasma gondii* [32] and *Pneumocystis carinii* [33], the membrane potential is not dependent on K^+^ but rather is proton driven. The permeability to K^+^in the plasma membrane is not predominant and it varies depending on the developmental stage of the parasite.We postulated previously that an inward rectifier K^+^ channel was involved in K^+^ uptake in *T. cruzi* trypomastigotes, dissipating the membrane potential generated by a plasma membrane located H^+^-ATPase and thereby facilitating its function in controlling pH_i_ [5]. In this work, we demonstrate that a gene encoding a protein with sequence homology to K^+^ channels is present in the *T. cruzi* genome (*TcCat*) and can complement yeast deficient in K^+^transporters, providing genetic evidence that it encodes a functional K^+^channel. In addition, we demonstrate by patch clamping giant liposomes containing recombinant TcCat that this channel has several distinct characteristics that differentiates it from mammalian inward rectifier K^+^(Kir)channels. Furthermore, this channel has the unexpected ability of translocating to different cellular compartments in response to environmental stress.

Examination of TcCat indicates that the only well conserved sequence of the protein compared with other K^+^ channels is its tetramerization domain, which is probably the reason why it was first annotated as a voltage-dependent K^+^ channel (TriTrypDB.org). The same domain is present in all voltage-dependent ion channels, including K^+^, Na^+^and Ca^2+^channels. BLASTP analysis of TcCat shows as only significant hits voltage-dependent K^+^channels from different organisms (e.g. E value: 3^−08^ to 6^−06^ for *Xenopus*). We need to consider that the overall sequence identity between ion channels is low. ClustalW alignments show that the identity between KcsA and *H. sapiens* Kv4.3 (both voltage-dependent K^+^channels) is only 23%. Same analysis indicates that TcCat and *H. sapiens* Kv4.1, and Kv4.3 are 19 and 20% identical, respectively. When inward-rectifier K^+^channels are compared, similar results are found, with 20% identity between *H. sapiens* Kir2.1 and KirBac1.1 and 23% identity between Kir2.1 and KirBac2.1. Moreover, when the comparison is established considering other evolutionary distant organisms, like *C. elegans*, the identity values obtained are always close to 20%. For other type of channels, like Na^+^and Ca^2+^channels, the conservation is even lower, underscoring the relevance of functional validation to establish the function of putative ion channels.

TcCat lacks the conserved K^+^ channel signature sequence [T-X-G-Y(F)-G] [20], which is compatible with the relatively low selectivity of the channel for K^+^ over Na^+^ and the possibility that TcCat can potentially transport both ions although the relative permeability ratio P_Na_/P_K_ is lower than the values previously reported for other non-selective cation channels [34], [35]. This is in agreement with results showing that either K^+^ or Na^+^ (at high concentrations) are important for pH_i_ control in trypomastigotes under acidic conditions [5]. Unfortunately, there are no structural data identifying amino acids that form the pore in non-selective cation channels and the sequence TLESW, recently identified as the selectivity filter for Na^+^channels [36], is not present in TcCat. The extracellular pore-forming region of TcCat has a short sequence (TFGADG in TcCat and TYGADG in TbCat and LmCat, Fig. S1) characterized by the presence of negatively charged amino acids, and a glycine residue, which could be involved in K^+^ selectivity, as occurs in HKT transporters from plants [37] and bacteria [38], and that is also conserved in the TbKHT1 K^+^ transporter of *T. brucei* [39]. This selectivity filter would be more similar to the low conserved pores described for some bacterial KirBac [40] or with the mutation in the pore of Kv3.1 in weaver mice that turns it into a non-selective cation channel [41], [42]. The presence of a distinct selectivity filter in TcCat could be important for the development of specific inhibitors with therapeutic potential against trypanosomes. In bloodstream trypomastigotes TcCat is exposed to the cell surface making it accessible to blockade by pharmacologic agents.

Previous investigators have used yeast strains carrying *trk1Δ* and *trk2Δ* deletions for complementation with inward-rectifying K^+^ channels from a variety of organisms [43]–[45]. However, it has been indicated that many inwardly rectifying K^+^ channels (as occurs with TcCat) are inhibited by high concentrations of external divalent cations and that to analyze heterologously expressed K^+^ channels in yeast it is desirable to reduce the concentration of external divalent cations. These conditions, however, favor the activity of the non-specific uptake system NSCI [46]. On the other hand, growth at the low pH required for mutants carrying the *trk1, trk2*, and *tok1* deletions used in this work inactivates NSC1 [25]. The complemented mutants obtained in this work will therefore provide a versatile genetic system for further studies of the assembly and composition of TcCat.

There is no previous electrophysiological description of the biophysical properties of ion channels in trypanosomatids. There are several limitations for the characterization of ion channels in motile unicellular organisms. Small size, irregular shape and active motility represent a problem for direct recording. The presence of a strong subpellicularcytoskeleton beneath the plasma membrane makes extremely hard to excise the patch and obtain a seal of suitable quality for single-channel recordings. The alternative of a cell-attached configuration is limited by the noise that the motility of the parasite introduces. We failed in many attempts to do direct patch-clamp in the parasites. Methods that decrease the motility like low temperature or use of actin-depolymerizing agents were considered but it can also be argued that they change the physiological conditions of the cell making the results obtained subject to discussion. Based on these facts we decided to use a reconstituted system in giant liposomes for ion channel characterization that has been extensively validated [19], [35], [47]–[58].

Although the mechanism by which the proteins are inserted in the liposomes is unknown, the orientation in which this occurs is not random. Reconstitution of acetylcholine receptors [47], glycine receptors [48], glutamate receptors [49], KcsA [52], [59] and KirBac1.1 [57] indicate that the proteins are oriented “right-side out”, meaning with the intracellular side facing the bath. The direction of the rectification observed for TcCat (Fig. 4B) suggests that this channel is also oriented with the intracellular side facing the bath.

The low conservation of the structure, particularly the sequence of the selectivity filter, can explain the functional differences observed in TcCat compared with other cation channels. Characterization of K^+^channels, although detailed and exhaustive in some cases, is mainly limited to bacterial (KcsA) and mammalian channels, with some particular cases of model organisms like *Drosophila* and *C. elegans*. In *Fasciola hepatica* [60]and *Dictyosteliumdiscoideum* [61] the presence of K^+^ channels with relative permeability ratio P_K_/P_Cl_ of 5 and 7, respectively, have been previously reported, suggesting that the selectivity of some channels in these organisms is not as high as for bacterial or mammalian K^+^channels.

Electrophysiological characterization of TcCat by patch clamping of giant liposomes indicates characteristics of K^+^ permeable channel with inward rectification characterized by non-linear current potential relationship for the single channel conductance. Therectification is weak, similar to what has been reported for KirBac1.1 and Kir1.1 [20], [57]. TcCat unitary conductance is significantly higher at negative than at positive potentials only in the presence of Mg^2+^, with no important differences in the outward currents suggesting a different mechanism of TcCat blockage by Mg^2+^. Although the residue responsible for the rectification in mammalian Kir channels (171D) is conserved in TcCat (Fig. S1C), it is followed by a positively charged amino acid (171His), which could potentially interfere with the binding of Mg^2+^ to the aspartic acid residue. Site-directed mutagenesis studies are in our future plans to address this and other structural properties.

Overall, the open probability did not show significant voltage-dependency. We have to mention that important differences were observed with different preparations. This could be explained by variations in the way that channels associate into clusters when reconstituted in liposomes. This type of behavior has been previously reported when purified KcsA was recorded in giant liposomes (52).

TcCat also differs from other inward rectifier K^+^ channels in its low selectivity for K^+^ over Na^+^, suggesting that it can transport either cation, and in its pharmacology. Blockers most commonly used for Kir channels are Ba^2+^ and Cs^+^ while TEA and 4-AP are known as inhibitors of Kv channels but have little effect on Kir channels [20]. TcCat was much less sensitive to Ba^2+^ than classical Kir channels (Ki 540–630 µM as compared to 13–390 µM for Kir2.x channels, [20]) and was insensitive to Cs^+^, when assayed in giant liposomes. Cs^+^, however, was as effective as Ba^2+^ in decreasing pH_i_ of intact trypomastigotes [5]. TcCat is not permeable to Ca^2+^, even more it can be blocked by it at similar concentration that we have previously reported as inhibitory for non-selective cation channels from *T. cruzi* epimastigotes membranes [35].

Although Ca^2+^usually does not block inward-rectifier channels, structural and functional differences observed in TcCat make it a non-canonical inward rectifier. KirBac1.1 is also a weak rectifier K^+^channel that has been demonstrated to show several atypical behaviors compared with mammalian Kirs like inhibition by Ba^2+^and Ca^2+^ [62], polyamine insensitivity [57] and blockage (instead of activation) by PIP_2_ [63]. In addition, 4-AP had inhibitory effect on TcCat total current at relatively low concentrations (1 µM).

It has been reported that although free-living prokaryotes have recognizable K^+^ channel genes, most but not all, parasitic prokaryotes have no K^+^ channel genes since they live in the K^+^-rich environment of their host cells [64]. The eukaryote *T. cruzi*, which has both intracellular and extracellular stages has solved the problem of having a K^+^ channel while in a K^+^-rich environment by sequestering it to an intracellular location in intracellular amastigotes. This sequestration starts very early during differentiation of trypomastigotes upon acid pH-stimulation. Interestingly, if the amastigotes are released as such to the extracellular medium, poor in K^+^, the channel reappears at the surface of the cells. Translocation of the channel to the surface also occurs in epimastigotes submitted to hyperosmotic stress suggesting a role for this channel in the recovery from this type of stress. Although a number of mechanisms are involved in the control of localization of K^+^channels in other cells [20], there is no precedent for the rapid translocation of TcCat that occurs when the cells are submitted to acidic pH (trypomastigotes), hyperosmotic (epimastigotes), or extracellular stress (amastigotes). Rapid phosphorylation/dephosphorylation changes could be involved in this translocation, as occurs with Kv4.2 [65] and ROMK [66]. The two-pore K^+^ channels K_2P_ 3.1 and K_2P_ 9.1 cell surface destination is also dependent on phosphorylation which regulates the interaction with 14.3.3 proteins [67]. Ιν αδδιτον, the localization of TcCat in the flagellar membrane of trypomastigotes suggests a role for this channel in the modulation of flagellar motility and sensing. In this regard, K^+^ channels are required to modulate the motility of ciliates and sperm cells [68] and Ca^2+^ channels located in the flagellar membrane of *T. brucei* are important for flagellar attachment and intracellular signaling [69].

In conclusion, we identified and characterized, at the molecular and biochemical levels, an novel inward-rectifier cation channel in *T. cruzi* with electrophysiological characteristics different from other Kir channels and that has the surprising ability to change its cellular localization when cells are exposed to different environmental stresses. In addition we have obtained yeast mutants that will provide a useful genetic system for studies of the assembly and composition of the channel and we demonstrated the feasibility of purifying a functional ion channel from *T. cruzi* after recombinant expression in bacteria.

## Materials and Methods

### Recombinant protein expression, purification and antibody generation

The entire open reading frame of *TcCat* was amplified by PCR with the primers 5′-CGGGATCCATGAGAAGGCGGGCCGTC-3′ and 3′-AACTGCAGTTAATGCGCTCTCCATATGTC -5′ introducing restriction sites for BamHI and PstI (*underlined*). PCR product was cloned into pGEM-T easy (Promega) and verified by automated sequencing. Cloned product was digested with restriction enzymes and ligated into pQE80L (Qiagen) expression vector. Expression of the recombinant protein in *E. coli* pLysS strain was induced with 0.5 mM isopropyl-β-D-thiogalactopyranoside (IPTG) overnight at 37°C. His-tagged recombinant protein was purified under denaturing conditions with His-Bind cartridges (Novagen). Purified product was separated by SDS-PAGE, stained with Coomasie blue and the corresponding band was excised from the gel and used as immunogen to obtain a rabbit polyclonal antibody against TcCat at CocalicoBiologicals, Inc (Reamstown, PA).

### Immunofluorescence and western blot analyses

For immunofluorescence microscopy, parasites were fixed in PBS, pH 7.4, with 4% paraformaldehyde, adhered to poly-lysine coverslips, and permeabilized for 3 min with PBS, pH 7.4, containing 0.3% Triton X-100. Permeabilized cells were treated for 30 min at room temperature with 50 mM NH_4_Cl and blocked overnight with 3% BSA in PBS pH 8.0. Purified polyclonal antibody against TcCat (dilution 1∶250) was incubated for 1 h at room temperature. Goat α-mouse and goat α-rabbit Alexa conjugated secondary antibodies (1∶2,000) were incubated for 1 h at room temperature. DNA-containing organelles were stained with 4′,6-diamidino-2-phenylindole (DAPI) (5 µg/ml). For TcCat immunolocalization in intracellular amastigotes, the cells were grown in coverslips and fixed at different times post-infection in cold methanol for 20 min. Immunolocalization in non-permeabilized parasites was done as described omitting the permeabilization step. A monoclonal antibody against *T. brucei* phosphate pyruvate dikinase (PPDK) (glycosomal marker) (a gift from Frédéric Bringaud, Université Bordeaux Segalen, France) was used as a permeabilization control. Differential interference contrast (DIC) and direct fluorescence images were obtained by using an Olympus IX-71 inverted fluorescence microscope with a PhotometrixCoolSnapHQ charge-coupled device camera driven by Delta Vision softWoRx3.5.1 (Applied Precision, Issaquah, WA). This same software was used to deconvolve and process the final images. The figures were built by using Adobe Photoshop 10.0.1 (Adobe System, Inc., San Jose, CA).

For western blot analysis, *T. cruzi* epimastigotes, amastigotes and trypomastigotes were collected by centrifugation at 1,600× *g* for 10 min, washed twice in PBS, pH 7.4, and resuspended in modified RIPA buffer (150 mM NaCl, 20 mM Tris-Cl pH 7.5, 1 mM EDTA, 1% SDS and 0.1% Triton X-100) containing protease inhibitor cocktail (2 mM EDTA, 2 mMphenylmethylsulfonyl fluoride (PMSF), 2 mMtosylphenylalanylchloromethyl ketone (TPCK), 0.1 mM*trans*-epoxysuccinyl-L-leucylamido(4-guanidino)butane (E64) and Sigma P8340 protease inhibitor cocktail, 1∶250). Total homogenate of each sample were separated by SDS-PAGE. Proteins were transferred onto nitrocellulose membranes and blocked overnight with 5% nonfat dry milk in PBS-0.1% Tween 20 (PBS-T). Blotting was done with α-TcCat (1∶5,000) and goat α-rabbit horseradish peroxidase conjugated antibodies (1∶20,000) for 1 h at room temperature and developed with ECL reagent. Membranes were stripped with 62.5 mMTris-HCl, pH 6.8, 2% SDS, 1% β-mercaptoethanol at 50°C for 30 min, extensively washed in PBS-T and incubated with monoclonal α-tubulin (Sigma) and goat α-mouse horseradish peroxidase conjugated antibodies (1∶10,000) as a loading control. Densitometric analysis of 4 independent experiments was performed with Alfa-Imager software.

#### Electrophysiological recordings

Multilamellar giant liposomes were prepared by mixing 50 µg of the DDM-purified TcCat with unilamellarasolectin vesicles in 10 mMKCl, 10 mMHepes-K, pH 7.4 in the presence of 5% ethylene glycol, as previously described [47] (see Additional Methods under Supplementary Information). After 4 h dehydration at 4°C, rehydration was done overnight in 10 mMKCl, 10 Hepes-K, pH 7.4, obtaining protein-containing giant liposomes ranging from 50 to 100 µm diameter. Multilamelar liposomes only containing the lipid mix were used as a control of leak currents.

For single channel recording by patch-clamp [70], aliquots (3–5 µl) of giant liposomes were placed into the recording chamber (RC-28, Warner Instruments Corporation, USA) and mixed with 0.5 mL of the buffer of choice for electrical recording (bath solution). Giga seals were formed on giant liposomes with glass-microelectrodes of 5–10 MΩ resistance. After sealing, withdrawal of the pipette from the liposome surface resulted in an excised patch. Current was recorded with an Axopatch 200B amplifier (Axon Instruments, Molecular Devices) at a gain of 100 mV/pA with a 1 kHz filter. The holding potential was applied to the interior of the patch pipette, and the bath was maintained at virtual ground (*V = V_bath_−V_pipette_*). The bath was grounded via an agar bridge and the junction potential was compensated when necessary. Recordings were analyzed off-line with pCLAMP10 software (Axon Instruments, Molecular Devices) and Microcal Origin 7.0 (Microcal Software, Inc., USA) software. All the experiments were conducted at room temperature.

### Osmotic stress

*T. cruzi* trypomastigotes and epimastigotes at log phase of growth (3 days) were collected at 1,600× *g* for 5 min, washed twice in PBS and resuspended in isosmotic buffer (64 mMNaCl, 4 mMKCl, 1.8 mM CaCl_2_, 0.53 mM MgCl_2_, 5.5 mM glucose, 150 mM D-mannitol, 5 mMHepes-Na, pH 7.4, 282 mosmol/L) at a cell density of 1×10^8^/ml. Aliquots of 5×10^6^ parasites were placed in tubes and 500 µl of either hyposmotic (64 mMNaCl, 4 mMKCl, 1.8 mM CaCl_2_, 0.53 mM MgCl_2_, 5.5 mM glucose, 50 mM D-mannitol, 5 mMHepes-Na, pH 7.4, 177 mosmol/L) or hyperosmotic buffer (64 mMNaCl, 4 mMKCl, 1.8 mM CaCl_2_, 0.53 mM MgCl_2_, 5.5 mM glucose, 500 mM D-mannitol, 5 mMHepes-Na, pH 7.4, 650 mosmol/L) were added. TcCat blockers were added at the indicated concentrations to the corresponding buffers. Cells were fixed at different times after osmotic stress by adding same volume of 8% paraformaldehyde in PBS, pH 7.4, and immunofluorescence analysis was performed as described before. Relative cell volume changes after osmotic stress were measured by light scattering method [71]. Briefly, the cells were washed twice in PBS and resuspended at a density of 4×10^8^ cells/ml in isosmotic buffer. Aliquots of 4×10^8^ parasites were distributed in 96 well plates and an appropriate volume of hyperosmotic buffer was added to reach a final osmolarity of 650 mosmol/L. Absorbance at 550 nm was monitored every 10 sec for 12 min. The results were normalized respect to the value of a 3 min pre-reading under isosmotic conditions.

To measure TcCat release after hyperosmotic stress, trypomastigotes and epimastigotes under osmotic stress were collected by centrifugation (1,600× *g* for 10 min) after 2 min of treatment. Supernatants were precipitated with 10% trichloroacetic acid for 1 h on ice. Precipitated proteins were collected by high-speed centrifugation (20,000× *g* for 20 min), washed and evaluated by western-blot analysis using anti-TcCat. Anti-tubulin was used as control to show that no parasite material was present in the supernatant, other than the released protein. Parasites overexpressing GFP were used as a control to rule-out lysis of the cells as a mechanism of release of TcCat (Fig. S7A).

## Supporting Information

Figure S1 **Conserved features for cation channels in trypanosomatids.** **A**. Multisequence amino acid alignment of putative cation channels in *T. cruzi* (TcCat) *T. brucei* (TbCat)and *L. major* (LmCat). Identical residues in the three species are *black shaded*, identical residues between two of them are *grey shaded*. Tetramerization domain (TcCat residues 5–73) and transmembrane domains (TM1 and TM2) are underlined. **B**. Amino acid alignment of conserved TcCat and *Homo sapiens* Kv4.3 tetramerization domain. Identical residues are *black shaded*, conserved substitutions are *grey shaded*. **C**. Amino acid alignment of inward-rectifier channels from *H. sapiens* (HsKir1.1), *E. coli* (KirBac1.1) and *T. cruzi* (tcru).
(TIF)

Figure S2 **TcCat is exposed at the cellular surface and co-localizes with plasma membrane markers.** TcCat immunolocalization in permeabilized vs non-permeabilized parasites. TcCat was detected with specific antibody (*green*). The glycosomal marker PPDK (*red*) was used as a permeabilization control both in permeabilized and non-permeabilized cells and is only detected in permeabilized cells.
(TIF)

Figure S3 **Loading control in the three stages of the parasite.** Coomassie blue staining of total protein homogenates from trypomastigotes (T), epimastigotes (E) and amastigotes (A) separated in a 10% SDS-PAGE gel. Prestained molecular weight markers (Invitrogen) are shown at the left.
(TIF)

Figure S4 **Outline of recombinant TcCat purification and reconstitution into liposomes suitable for electrophysiology.** **A**. Recombinant protein induction in bacteria. The complete ORF for TcCat was amplified by PCR and cloned into pQE80L expression vector. *E. coli* plysS strain transformed with the vector were induced with 0.5 mM IPTG overnight at 37°C and aliquots of the cells were separated by SDS-PAGE and Coomassie-blue stained. pLys: non-transformed bacteria, E: pLysS bacteria transformed with the empty vector, NI: non-induced bacteria transformed with TcCat-pQE80L vector, I: induced pLysS containing TcCat-pQE80L vector. **B**. TcCat recombinant protein purification in the presence of DDM at micellar concentration was verified by western blot analysis with monoclonal anti-His tag antibody (anti-penta His tag, Qiagen). Lanes, 1: molecular weight marker, 2: non-induced bacteria, 3: induced bacteria, 4: supernatant 1 (see Text S1), 5: pellet 1 (P1), 6: supernatant 2 (S2), 7: flow-through Ni-agarose column, 8: purified protein after dialysis, 9: purified TcCat incorporated into unilamellarasolectin vesicles, 10: TcCat recombinant protein purified under denaturing conditions. **C**. Left panel: Cy5-TcCat incorporated into unilamellar vesicles was verified by microscopy (DIC-red, *top panel*) and western blot analysis (*bottom panel*) with anti-TcCat antibody using increasing amount of protein (2.5, 5 and 10 µg from left to right lanes). *Right panel*: empty unilamellar vesicles were used as a control for microscopy analysis. **D**. Unilamellar vesicles containing purified TcCat were fused with empty asolectinunilamellar vesicles to produce multilamellar giant liposomes (**E**) that were used for electrophysiological recordings.
(TIF)

Figure S5 **Evaluation of TcCat purification.** BL21 codon plus bacteria containing the empty vector pQE80L (**A**) or TcCat-pQE80L (**B**) were induced and purified as described above. Aliquots of key steps of the purification were taken, electrophoresed by SDS-PAGE and Coomassie blue stained. NI: non-induced bacteria, I: induced bacteria, H: homogenate, F: flow-through Ni^2+^-agarose column, W: wash Ni^2+^-agarose column, E: elution of the purified proteins. Bands identified A to D were analyzed by mass spectrometry. Arrow indicates the expected size for TcCat. Two different molecular weight markers were used to have a better estimation of the sizes.
(TIF)

Figure S6 **Effect of divalent cations on TcCat currents.** **A**. Representative current-voltage relationship obtained from empty liposomes (*black squares*) or TcCat-containing liposomes (*red circles*). The difference in the current observed in both situations indicate that the leak current through asolectin vesicles is small and that TcCat forms active ion channels. **B**. Ba^2+^ effect on the asolectin leakage. Normalized currents (respect to the current in the absence of the divalent cation) in the presence of increasing concentrations of BaCl_2_. Empty liposomes (*black circles*) or TcCat liposomes (*black squares*) were recorded at −80 mV (*upper panel*) or +80 mV (*lower panel*). *Red lines* correspond to the fitting of the data to an exponential decay function. **C, E**. Concentration-dependent inhibition of TcCat currents by Ba^2+^ (**C**) or Ca^2+^(**E**). (**D, F**) Total current of the seal was normalized respect to the values recorded in the absence of Ba^2+^ (**D**) or Ca^2+^(**F**) at different voltages.
(TIF)

Figure S7 **TcCat release to the extracellular medium.** Western blot analysis of TcCat in supernatants of trypomastigotes (**A**) and epimastigotes (**B**) under osmotic stress. Iso: isosmotic buffer; Hypo: hyposmotic buffer; Hyper: hyperosmotic buffer. Anti-tubulin antibody and anti-GFP were used as controls for lysis of the cells.
(TIF)

Figure S8 **Effect of tubulin de-polimerization agents on TcCat translocation.** TcCatimmunolocalization in *T. cruzi* epimastigotes (**A**) and trypomastigotes (**B**) under isosmotic or hyperosmotic conditions. Parasites were pre-incubated for 10 min at 37°C with 500 µMtrifluralin or 10 µMchloralin before the osmotic stress, where indicated. TcCat was detected with purified specific antibody and secondary anti-rabbit Alexa-488 conjugated (*green*). DNA was stained with DAPI (*blue*). Bars = 10 µm.
(TIF)

Table S1 **Mass spectrometry identification of proteins co-purified with TcCat.** Bands labeled A to D in Figure S5B were trypsin-digested and identified by mass spectrometry. Identification numbers, total scores and number of peptides are presented. Proteins identified below a 1% false protein discovery rate were considered significant.
(DOCX)

Text S1 **Topology analysis, equations, yeast culture, yeast constructs and complementation, verification of TcCat expression in yeast, TcCat recombinant protein purification and reconstitution into liposomes, mass spectrometry and references.**
(DOCX)
