# Supplementary figures and images for: Molecular and Electrophysiological Characterization of a Novel Cation Channel of Trypanosoma cruzi
Source: PLoS Pathog. 2012 Jun 7;8(6):e1002750. doi: 10.1371/journal.ppat.1002750 (PMC3369953; doi:10.1371/journal.ppat.1002750)

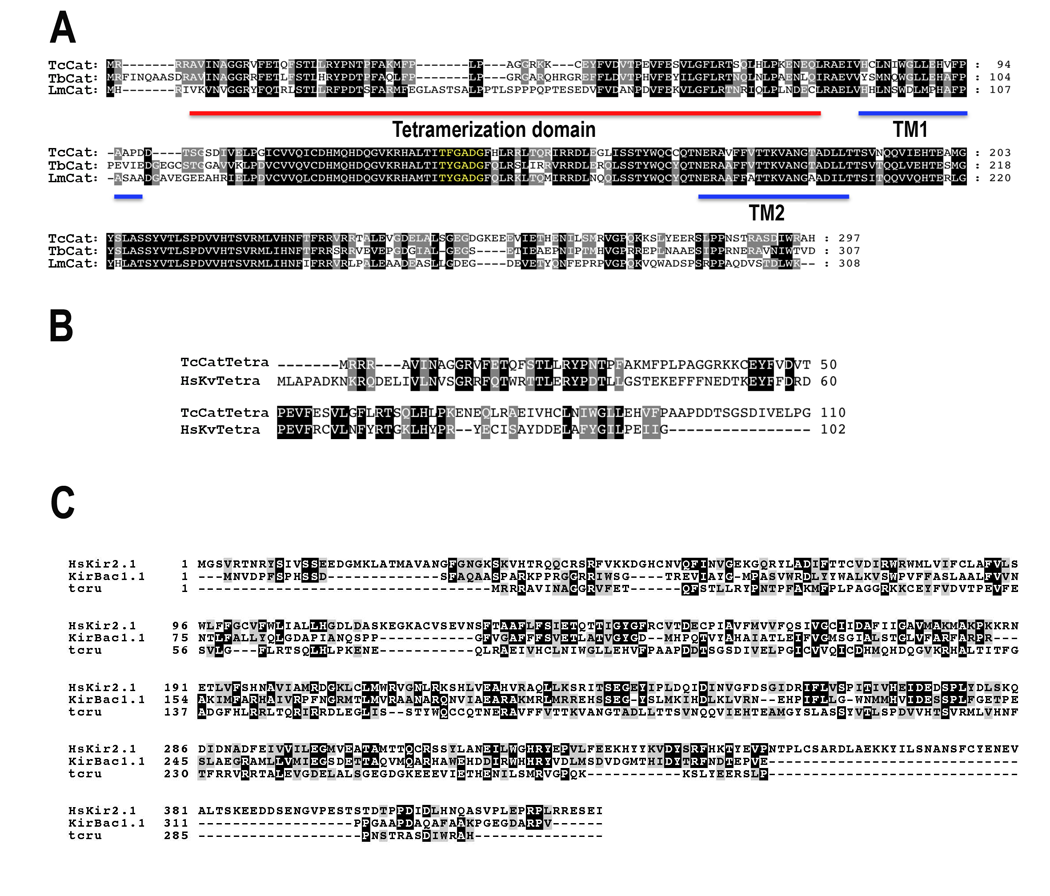

Supplement: Figure S1 — Conserved features for cation channels in trypanosomatids. A. Multisequence amino acid alignment of putative cation channels in T. cruzi (TcCat) T. brucei (TbCat)and L. major (LmCat). Identical residues in the three species are black shaded, identical residues between two of them are grey shaded. Tetramerization domain (TcCat residues 5–73) and transmembrane domains (TM1 and TM2) are underlined. B. Amino acid alignment of conserved TcCat and Homo sapiens Kv4.3 tetramerization domain. Identical residues are black shaded, conserved substitutions are grey shaded. C. Amino acid alignment of inward-rectifier channels from H. sapiens (HsKir1.1), E. coli (KirBac1.1) and T. cruzi (tcru). (TIF) [file ppat.1002750.s001.tif]

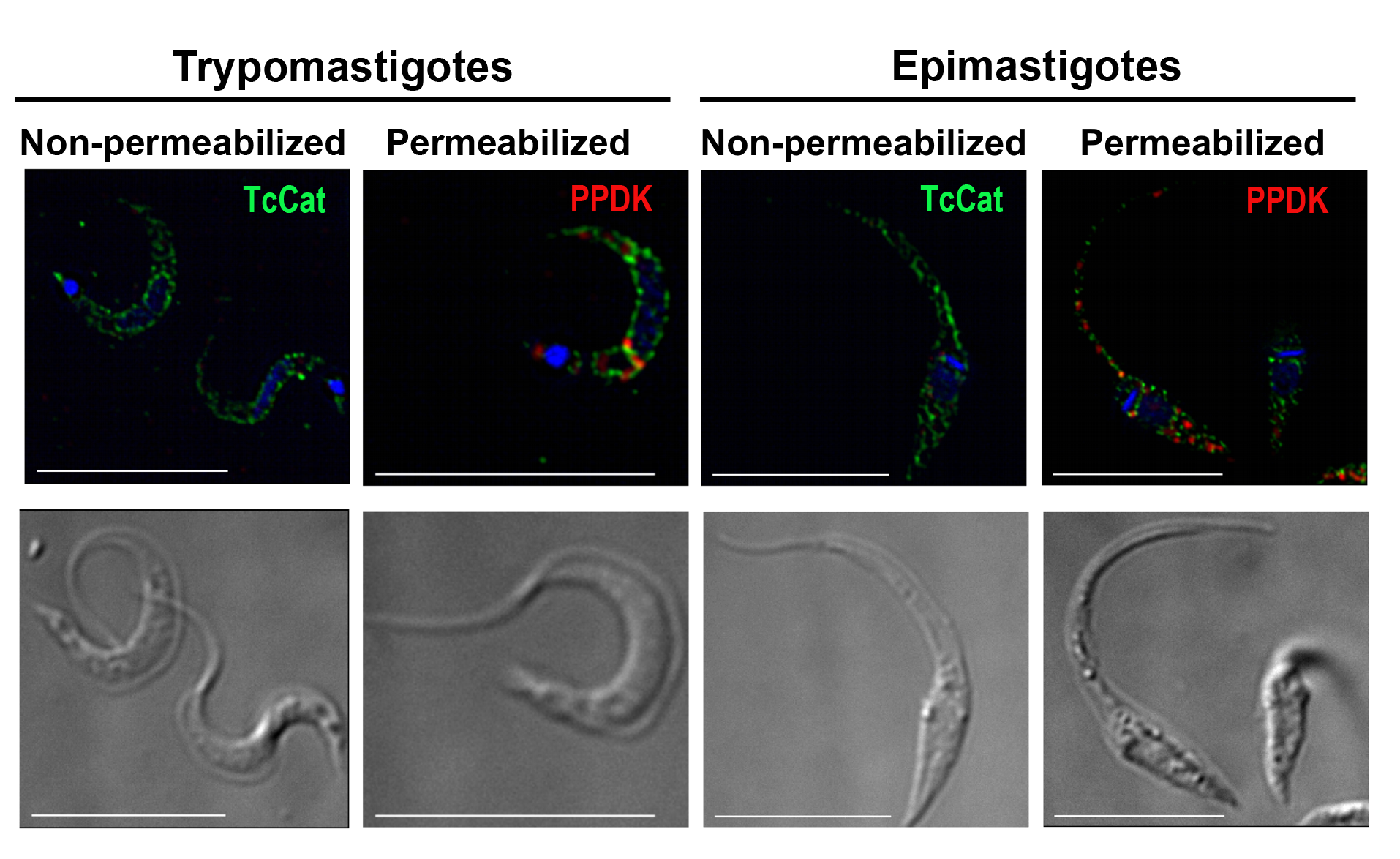

Supplement: Figure S2 — TcCat is exposed at the cellular surface and co-localizes with plasma membrane markers. TcCat immunolocalization in permeabilized vs non-permeabilized parasites. TcCat was detected with specific antibody (green). The glycosomal marker PPDK (red) was used as a permeabilization control both in permeabilized and non-permeabilized cells and is only detected in permeabilized cells. (TIF) [file ppat.1002750.s002.tif]

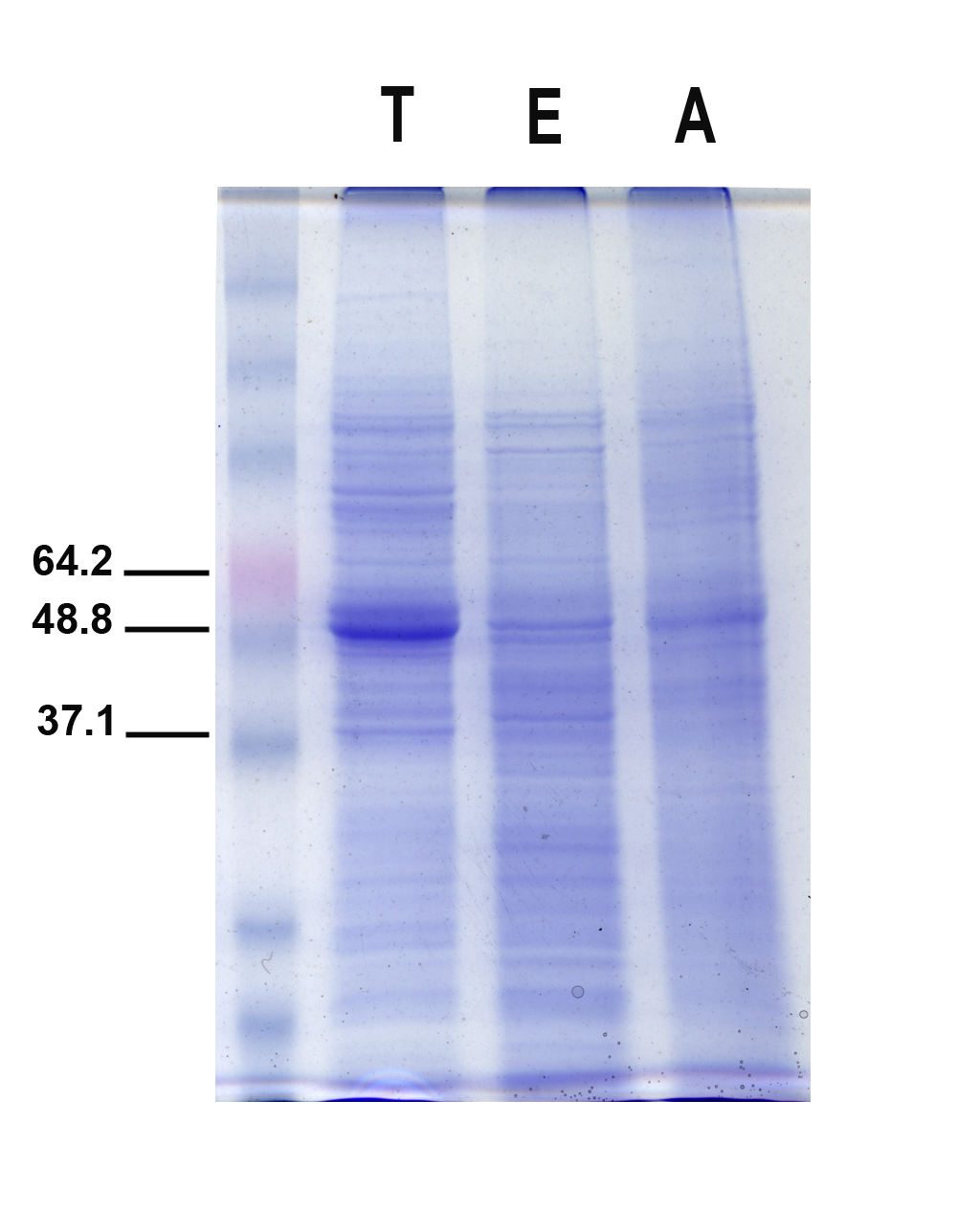

Supplement: Figure S3 — Loading control in the three stages of the parasite. Coomassie blue staining of total protein homogenates from trypomastigotes (T), epimastigotes (E) and amastigotes (A) separated in a 10% SDS-PAGE gel. Prestained molecular weight markers (Invitrogen) are shown at the left. (TIF) [file ppat.1002750.s003.tif]

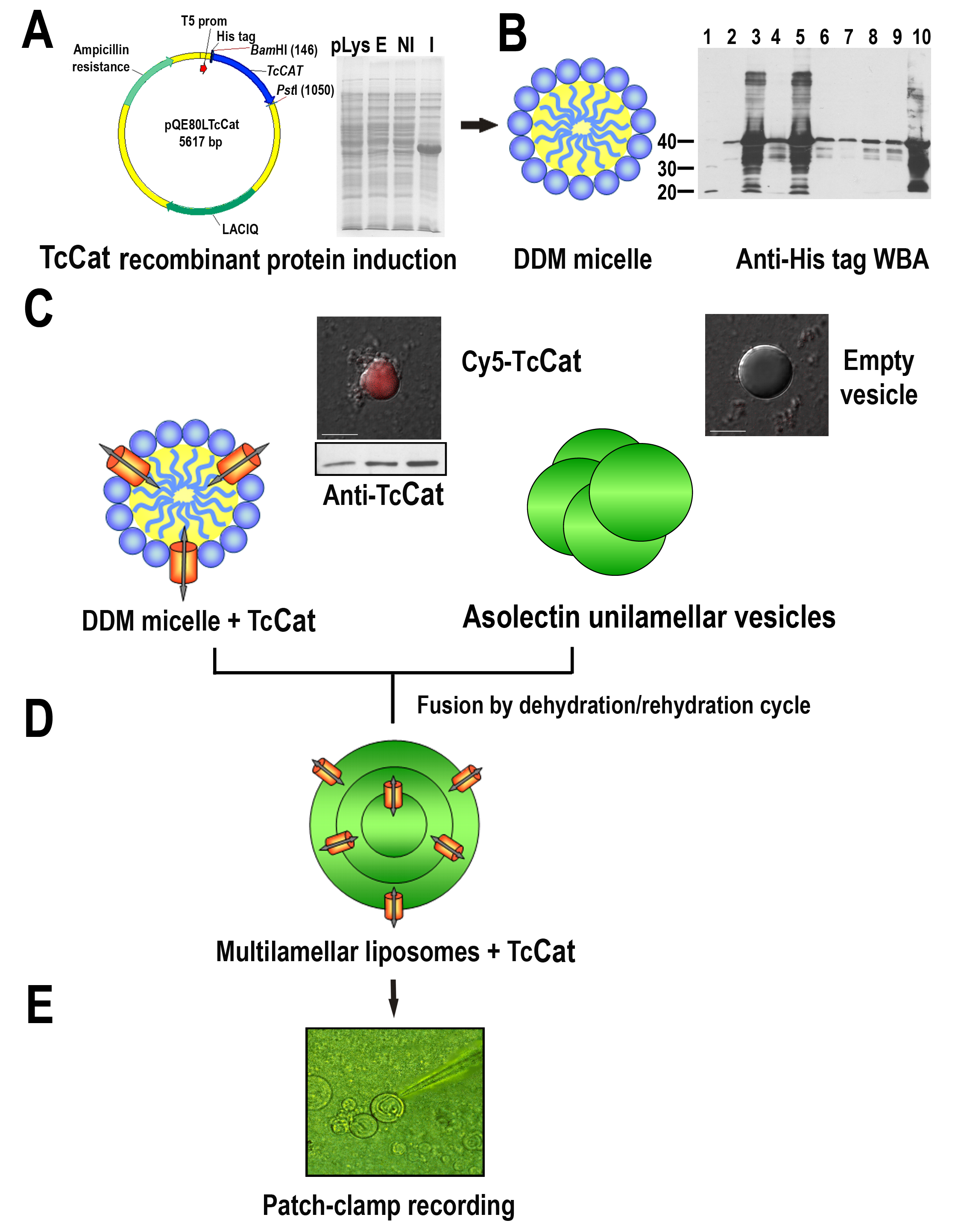

Supplement: Figure S4 — Outline of recombinant TcCat purification and reconstitution into liposomes suitable for electrophysiology. A. Recombinant protein induction in bacteria. The complete ORF for TcCat was amplified by PCR and cloned into pQE80L expression vector. E. coli plysS strain transformed with the vector were induced with 0.5 mM IPTG overnight at 37°C and aliquots of the cells were separated by SDS-PAGE and Coomassie-blue stained. pLys: non-transformed bacteria, E: pLysS bacteria transformed with the empty vector, NI: non-induced bacteria transformed with TcCat-pQE80L vector, I: induced pLysS containing TcCat-pQE80L vector. B. TcCat recombinant protein purification in the presence of DDM at micellar concentration was verified by western blot analysis with monoclonal anti-His tag antibody (anti-penta His tag, Qiagen). Lanes, 1: molecular weight marker, 2: non-induced bacteria, 3: induced bacteria, 4: supernatant 1 (see Text S1), 5: pellet 1 (P1), 6: supernatant 2 (S2), 7: flow-through Ni-agarose column, 8: purified protein after dialysis, 9: purified TcCat incorporated into unilamellarasolectin vesicles, 10: TcCat recombinant protein purified under denaturing conditions. C. Left panel: Cy5-TcCat incorporated into unilamellar vesicles was verified by microscopy (DIC-red, top panel) and western blot analysis (bottom panel) with anti-TcCat antibody using increasing amount of protein (2.5, 5 and 10 µg from left to right lanes). Right panel: empty unilamellar vesicles were used as a control for microscopy analysis. D. Unilamellar vesicles containing purified TcCat were fused with empty asolectinunilamellar vesicles to produce multilamellar giant liposomes (E) that were used for electrophysiological recordings. (TIF) [file ppat.1002750.s004.tif]

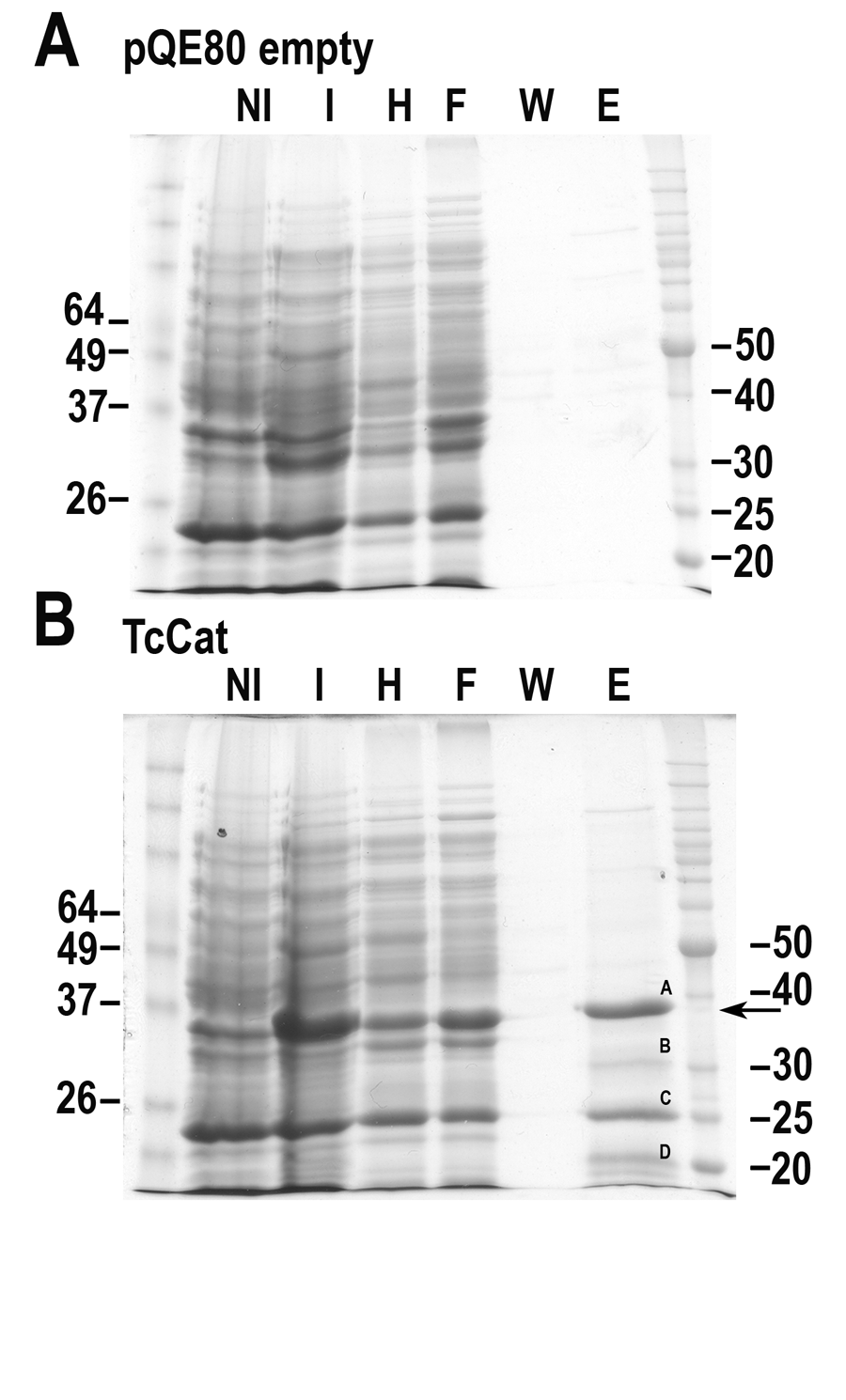

Supplement: Figure S5 — Evaluation of TcCat purification. BL21 codon plus bacteria containing the empty vector pQE80L (A) or TcCat-pQE80L (B) were induced and purified as described above. Aliquots of key steps of the purification were taken, electrophoresed by SDS-PAGE and Coomassie blue stained. NI: non-induced bacteria, I: induced bacteria, H: homogenate, F: flow-through Ni2+-agarose column, W: wash Ni2+-agarose column, E: elution of the purified proteins. Bands identified A to D were analyzed by mass spectrometry. Arrow indicates the expected size for TcCat. Two different molecular weight markers were used to have a better estimation of the sizes. (TIF) [file ppat.1002750.s005.tif]

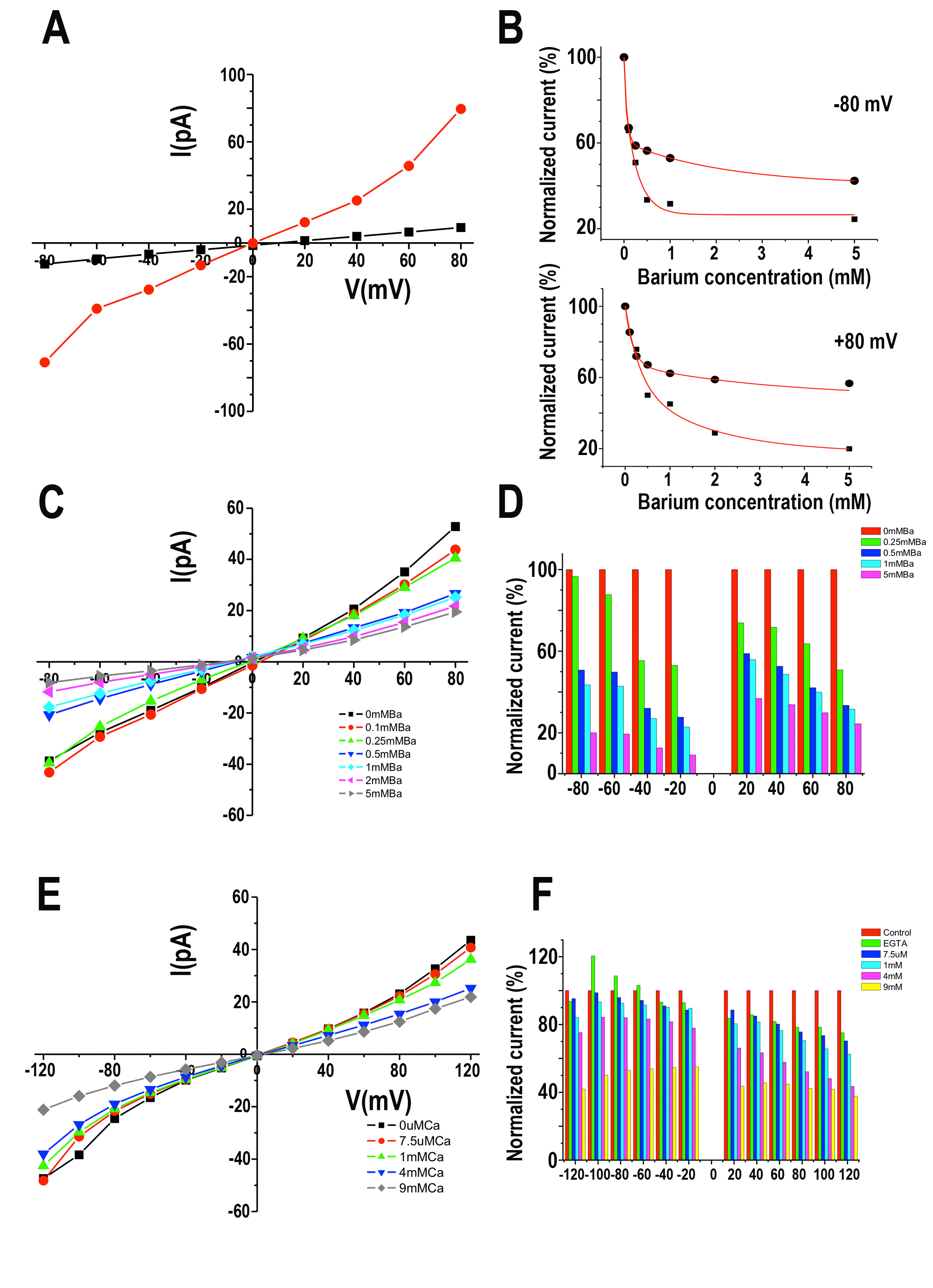

Supplement: Figure S6 — Effect of divalent cations on TcCat currents. A. Representative current-voltage relationship obtained from empty liposomes (black squares) or TcCat-containing liposomes (red circles). The difference in the current observed in both situations indicate that the leak current through asolectin vesicles is small and that TcCat forms active ion channels. B. Ba2+ effect on the asolectin leakage. Normalized currents (respect to the current in the absence of the divalent cation) in the presence of increasing concentrations of BaCl2. Empty liposomes (black circles) or TcCat liposomes (black squares) were recorded at −80 mV (upper panel) or +80 mV (lower panel). Red lines correspond to the fitting of the data to an exponential decay function. C, E. Concentration-dependent inhibition of TcCat currents by Ba2+ (C) or Ca2+(E). (D, F) Total current of the seal was normalized respect to the values recorded in the absence of Ba2+ (D) or Ca2+(F) at different voltages. (TIF) [file ppat.1002750.s006.tif]

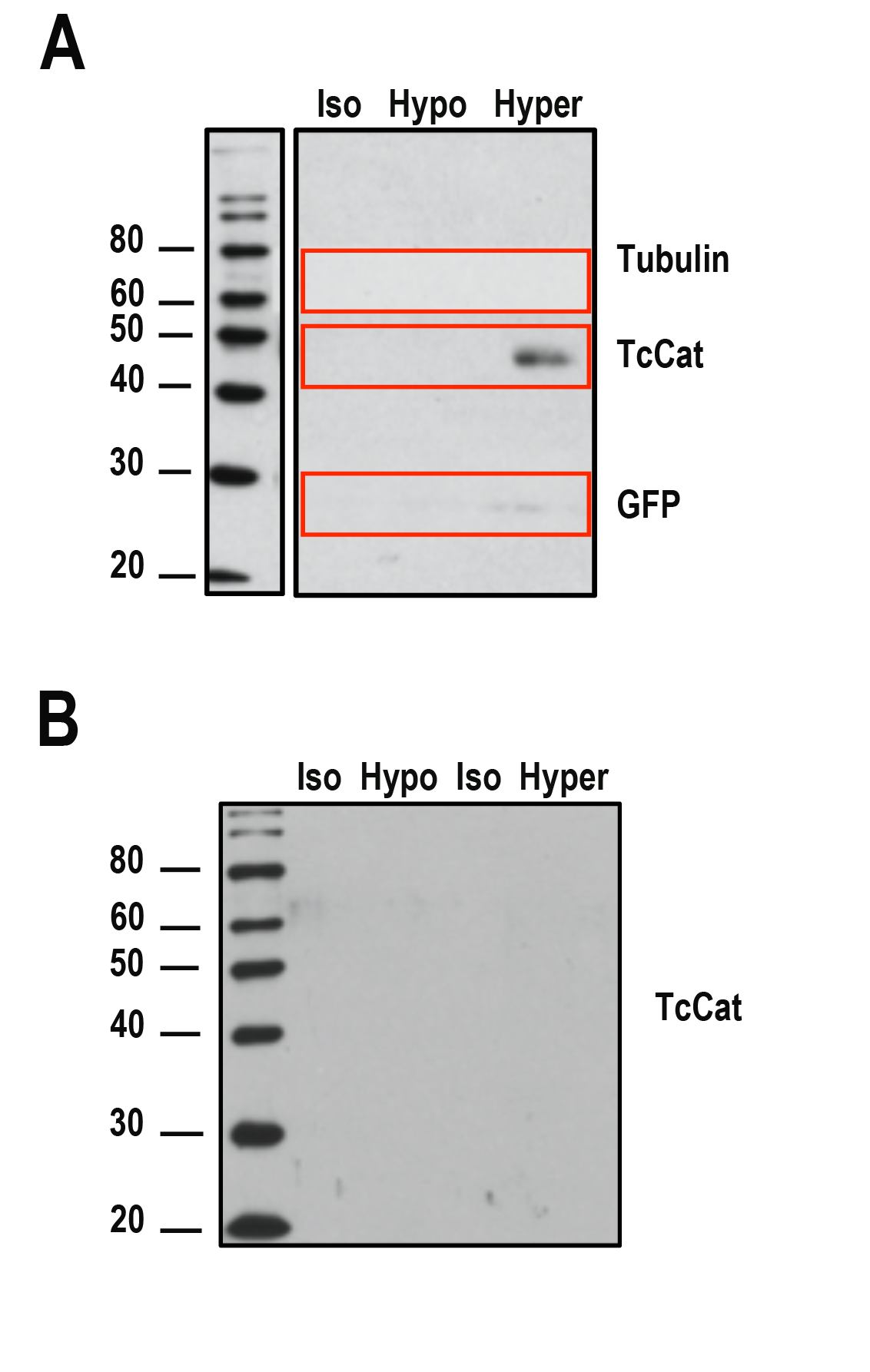

Supplement: Figure S7 — TcCat release to the extracellular medium. Western blot analysis of TcCat in supernatants of trypomastigotes (A) and epimastigotes (B) under osmotic stress. Iso: isosmotic buffer; Hypo: hyposmotic buffer; Hyper: hyperosmotic buffer. Anti-tubulin antibody and anti-GFP were used as controls for lysis of the cells. (TIF) [file ppat.1002750.s007.tif]

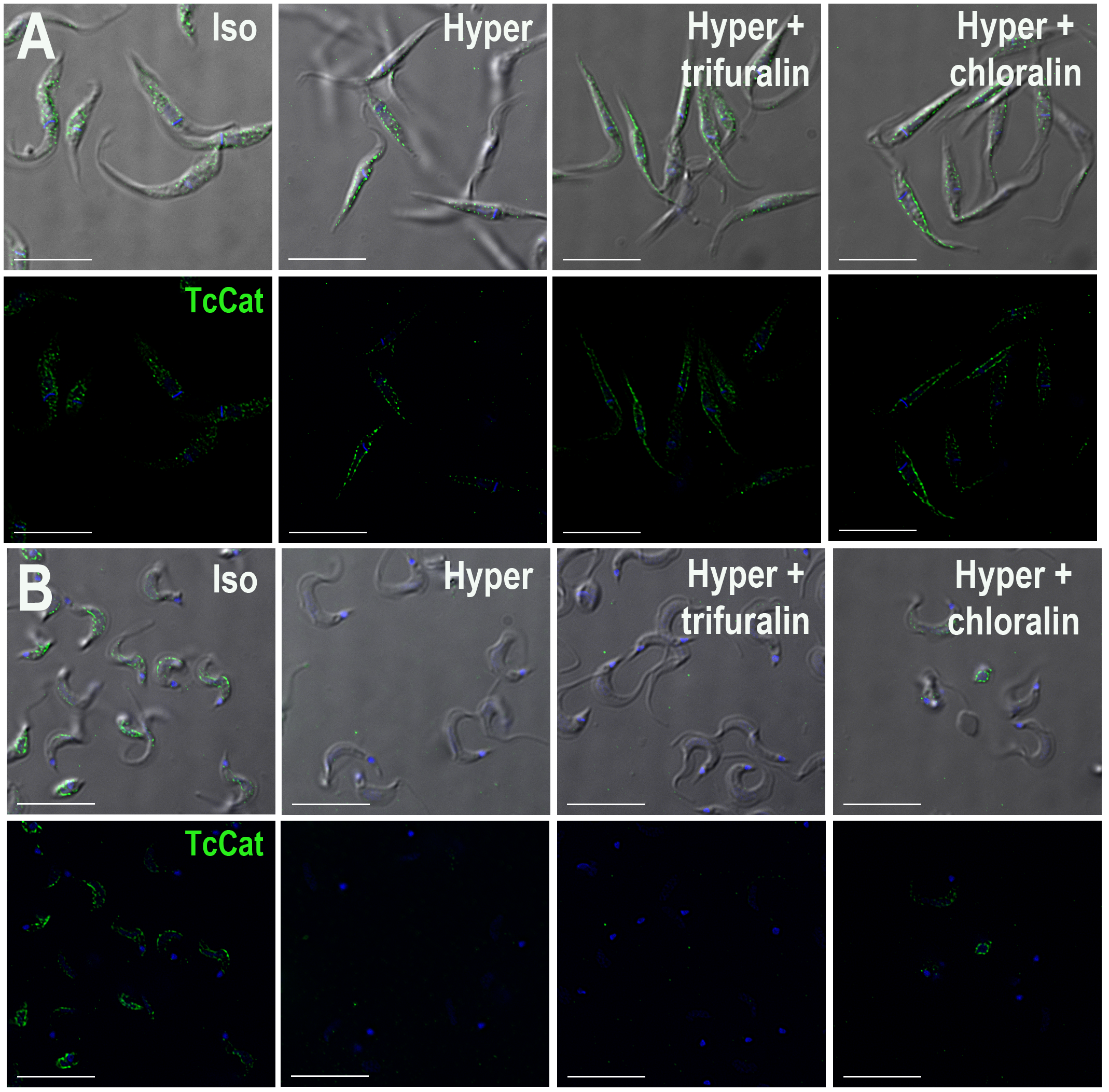

Supplement: Figure S8 — Effect of tubulin de-polimerization agents on TcCat translocation. TcCatimmunolocalization in T. cruzi epimastigotes (A) and trypomastigotes (B) under isosmotic or hyperosmotic conditions. Parasites were pre-incubated for 10 min at 37°C with 500 µMtrifluralin or 10 µMchloralin before the osmotic stress, where indicated. TcCat was detected with purified specific antibody and secondary anti-rabbit Alexa-488 conjugated (green). DNA was stained with DAPI (blue). Bars = 10 µm. (TIF) [file ppat.1002750.s008.tif]
