# Supplementary material for: Molecular and Electrophysiological Characterization of a Novel Cation Channel of Trypanosoma cruzi
Source: PLoS Pathog. 2012 Jun 7;8(6):e1002750. doi: 10.1371/journal.ppat.1002750 (PMC3369953; doi:10.1371/journal.ppat.1002750)
Supplement: Table S1 — Mass spectrometry identification of proteins co-purified with TcCat. Bands labeled A to D in Figure S5B were trypsin-digested and identified by mass spectrometry. Identification numbers, total scores and number of peptides are presented. Proteins identified below a 1% false protein discovery rate were considered significant. (DOCX) [file ppat.1002750.s009.docx]

**Table S1. Mass spectrometry identification of proteins co-purified with TcCat.**

| Band | Sequence Id | Sequence name | Protein weight (kDa) | Total score | Total peptides |
| --- | --- | --- | --- | --- | --- |
| A | gi\|71653718\|ref\|XP_815492.1\| | potassium voltage-gated channel [*Trypanosoma cruzi* strain CL Brener] | 33.49801794 | 2517.35 | 52 |
| B | gi\|157156604\|ref\|YP_001463444.1\| | tagatose-bisphosphate aldolase [*Escherichia coli* E24377A] | 30.81858324 | 389.67 | 12 |
|  | gi\|170650838\|ref\|YP_001740012.1\| | beta-lactamase TEM [*Escherichia coli* SMS-3-5] | 31.47718146 | 263.05 | 4 |
|  | gi\|157155478\|ref\|YP_001463603.1\| | polysaccharide deacetylase domain-containing protein [*Escherichia coli* E24377A] | 33.10296912 | 197.31 | 4 |
|  | gi\|117622454\|ref\|YP_851367.1\| | 30S ribosomal protein S2 [*Escherichia coli* APEC O1] | 26.70879633 | 137.59 | 4 |
|  | gi\|157156683\|ref\|YP_001464204.1\| | xanthine dehydrogenase subunit XdhB [*Escherichia coli* E24377A] | 31.50907688 | 104.27 | 2 |
|  | gi\|16129422\|ref\|NP_415980.1\| | N-hydroxyarylamine O-acetyltransferase [*Escherichia coli* str. K-12 substr. MG1655] | 32.23614891 | 79.11 | 2 |
| C | gi\|16128327\|ref\|NP_414876.1\| | thiogalactoside acetyltransferase [*Escherichia coli* str. K-12 substr. MG1655] | 22.76645709 | 268.71 | 7 |
|  | gi\|170682300\|ref\|YP_001742476.1\| | galactoside O-acetyltransferase [*Escherichia coli* SMS-3-5] | 23.10568031 | 231.84 | 6 |
| D | gi\|157160635\|ref\|YP_001457953.1\| | hypothetical protein EcHS_A1231 [*Escherichia coli* HS] | 21.18067328 | 146.36 | 4 |
