# Supplementary material for: Molecular and Electrophysiological Characterization of a Novel Cation Channel of Trypanosoma cruzi
Source: PLoS Pathog. 2012 Jun 7;8(6):e1002750. doi: 10.1371/journal.ppat.1002750 (PMC3369953; doi:10.1371/journal.ppat.1002750)
Supplement: Text S1 — Topology analysis, equations, yeast culture, yeast constructs and complementation, verification of TcCat expression in yeast, TcCat recombinant protein purification and reconstitution into liposomes, mass spectrometry and references. (DOCX) [file ppat.1002750.s010.docx]

**Text S1. Additional methods**

**Topology analysis**

TcCat topology was analyzed with TopPred2 (putative cut-off 0.6 and certain cut-off 1.0). Four putative transmembrane domain were predicted as follows:

Candidate membrane-spanning segments:

Helix Begin - End Score Certainity

1 44 - 64 0.701 Putative

2 77 - 97 0.820 Putative

3 100 - 120 0.903 Putative

4 169 - 189 0.626 Putative

Although the prediction for segments 2 and 3 has a higher score, the position of segment 3 does not allow the presence of a pore domain in between the segments 2 and 3. Comparison with the TM domain of other channels with two TM domains as well as the minimum critical distance necessary between TM domain indicate that segments 2 and 3 cannot correspond to TM1 and TM2 in TcCat. Topology prediction by TMPred also predicts a TM domain from amino acids 77 to 97. Based on this analysis we proposed the topology shown in Figure S1.

**Equations**

The TcCat single channel chord conductance (γ) was calculated at +80 and -80 mV following the equation:

*γ= i/V*

where *i* is the single channel current and *V* is the applied voltage. The unitary currents plotted were obtained as the peak of the histogram of the current for each applied voltage. The values in the graph represent the average of the peak of the histogram for single channel recordings ± SEM.

The open probability was calculated with Clampfit software using the option of channel search in recordings under symmetrical conditions in the absence of Mg^2+^, at different applied voltages in n = 14 independent experiments.

The theoretical reversal potential for each ion (*V_rev_*) was obtained according with the Nerst equation:

*V_rev_= RT/zF ln [X]_pip_/[X]_bath_*

R is the gas constant, T is the temperature in Kelvin scale, z is the valence of the ion, *[X]_pip_* is the ion concentration in the pipette and *[X]_bath_* is the concentration of the ion in the bath solution for an inside-out excised patch configuration.

Relative permeability ratio for chloride was calculated according to the derived equation from Goldman-Hodgkin-Katz (G-H-K) current equation:

*P_K_/P_Cl_= [Cl]_bath_-exp^(FV/RT)^ –[Cl]_pip_/ exp^(FV/RT)^[K]_bath_-[K]_pip_*

*P_K_* represents the permeability to K^+^, *P_Cl_* is the permeability to chloride, F is the Faraday constant, *R* is the gas constant, *T* is the absolute temperature and *V* is the experimental shift in the reversal potential.

The relative permeability ratio for monovalent cations respect to K^+^ in the absence of Mg^2+^ was calculated by the bi-ionic equation resultant of the derived G-H-K current equation:

*P_X_/ P_K_ = ([K]_bath_ exp ^(FV/RT)^ – [K]_pip_/ exp^(FV/RT)^ [X]_bath_*

where *P_X_* is the cation X permeability, *P_K_* is K^+^ permeability and *[X]_bath_* represents the monovalent cation concentration in the bath solution.

Based on the inhibition of the currents in the presence of Ba^2+^ or Ca^2+^, the inhibition constant (K_i_) for blockage by divalent cations was calculated according to the binding equation [1] for each voltage:

*i = i_o_ / (1 + A/K_i_)*

Where *i* is the total current in the patch without blocker, *i_o_* is the current after divalent ion addition and *A* is the divalent cation concentration.

**Yeast culture, yeast constructs and complementation**

*Saccharomyces cerevisiae* PLY232 (wild-type) and PLY246 (*trk1Δ trk2Δ* and *tok1Δ null* mutants) strains were kindly provided by Dr. Per O. Ljungdahl (Ludwig Institute for Cancer Research, Sweden) [2]. Wild type cells were maintained at 30°C in standard YPD medium and the mutants were supplemented with 50 mM KCl pH 5.8. *TcCat* ORF was amplified by PCR with forward and reverse primers 5’-CGGGATCCACCATGGGAAGGCGGGCCGTC-3’ and 3’-GGAATTCTTAATGCGCTCTCCATATGTTCG-5’ in order to introduce restriction sites for BamHI and EcoRI as well as a Kozac sequence, necessary for optimal expression in yeast. PCR product was cloned into ZeroBlunt TOPO cloning vector and verified by sequencing. The product was digested and ligated into pYES2 expression vector and wild type and mutant yeasts were transformed following manufacturer protocol. Transformed cells were selected in synthetic minimal defined medium without uracil, pH 5.8 (SC ura (-) medium) supplemented with 100 mM KCl to maintain the mutant under viable conditions. Positive clones were confirmed by PCR and expression of *TcCat* was induced switching the carbon source from 2% rafinose to 2% galactose. Complementation studies were done seeding serial dilutions of the complemented mutants in SC ura(-)-galactose agar plates without KCl added, keeping them at 30°C for 3 to 5 days. Wild-type strains transformed with *TcCat* or with the empty vector were used as a control.

**Verification of *TcCat* expression in yeast**

For western blot analysis, after expression induction, the cells were collected at 1,500 x g for 5 min at 4°C, washed in ice-cold water and resuspended in breaking buffer (50 mM sodium phosphate, pH 7.4, 1 mM EDTA, 1 mM phenylmethylsulfonyl fluoride (PMSF) in a volume to obtain an OD_600_ of 50. An equal volume of acid-washed glass beads was added and repeated vortex mixing broke the cells. The samples were then centrifuged at 16,000 x *g* for 5 min at 4°C and the supernatant containing the proteins was collected and analyzed by SDS-PAGE. After transfer to nitrocellulose, the membranes were incubated with α-TcCat (1:5,000) and goat α-rabbit horseradish peroxidase conjugated antibodies (1:20,000) for 1 h at room temperature and developed with ECL reagent. Immunofluorescence of yeast was performed as previosly described previously [3]. TcCat localization was evaluated with the polyclonal specific antibody (dilution 1:250). Permeabilization was assessed with a monoclonal antibody (8B1 clone, Molecular Probes) against the 69 kDa sub-unit of the vacuolar H^+^-ATPase (1:250).

**TcCat recombinant protein purification and reconstitution into liposomes**

The complete ORF of the channel was successfully expressed in bacteria (Fig. S3A), and purified in the presence of detergent according to Molina et al. [4] with minor modifications. Briefly, after induction of expression, bacteria containing *TcCat* or the empty vector pQE80L were pelleted at 2,600 *g* for 10 min at 4 ºC, washed in 1% NaCl and resuspended in lysozyme buffer (20 mM Hepes-K, pH 7.5, 0.45 M sucrose, 8 mM EDTA, 0.75 mM PMSF, and 0.4 mg/ml lysozyme). After 1 h incubation on ice, the mixture was sonicated in an ice bath for 2 min at 30% amplitude (Branson Digital Sonifier S250D) and centrifuged at 100,000 *g* for 1 h. An aliquot of the supernatant (S1) was taken for further analysis. The membrane-containing pellet (P1) was resuspended in 20 mM Hepes-K, pH 7.5, 100 mM KCl, 10 mM imidazole, 0.75 mM PMSF, and 10 mM dodecyl-β-D-maltoside (DDM) and incubated for 1 h at room temperature. The supernatant (S2) obtained after centrifuging for 1 h at 100,000 *g* was mixed with 5 ml Ni^2+^-NTA agarose beards (Qiagen) and incubated overnight at 4 ºC, packed in a column and washed with 20 mM Hepes-K, pH 7.5, 100 mM KCl, 10 mM imidazole, 0.75 mM PMSF, and 1 mM DDM until Bradford protein assay was below detection limit. The protein bound to the matrix was eluted with 20 mM Hepes-K, pH 7.5, 100 mM KCl, 1 mM imidazole, 0.75 mM PMSF, and 1 mM DDM and dialyzed overnight at 4 ºC against the same buffer without imidazole. The purified protein was quantified by the bicinchoninic acid (BCA) assay and the obtained yield was 1-2 mg of DDM solubilized protein/L of bacterial culture. The His-tagged TcCat was recovered as DDM-solubilized protein and transferred to unilamellar asolectin vesicles, as it could be verified by western blot analysis with anti His-tag antibody (Fig. S4B, lanes 1-10), anti TcCat antibody (Fig. S4C, *left panel, bottom*) and by microscopy of Cy5- labeled TcCat incorporated in lipid vesicles (Fig. S4C, *left panel top*). Four independent purifications showed similar results, with an estimated yield of 1-2 mg of purified protein reconstituted into unilamellar liposomes. Unilamellar vesicles containing TcCat were fused with asolectin vesicles by dehydration-rehydration cycles and the final giant multilamellar liposomes were used for single-channel patch-clamp experiments. A total of 89 giga-seals were obtained from independent preparations (4 protein purifications and 3 liposome preparations). 73% of them (65 seals) showed activity indicating that the purification and reconstitution method was highly reliable. Of those with activity, 90% presented adequate noise-signal ratio and stability for further analysis. The results of the electrophysiological characterization of TcCat correspond to a total of 59 analyzed giga-seals.

**Mass spectrometry**

*E.coli* BL21(DE3) codon plus were transformed with pQE80 plasmid without insert and selected with 100 µg/ml ampicillin. Colonies were screened by PCR with specific primers for the vector backbone forward 5’-ATTCAATTGTGAGCGGATAAC-3’ and reverse 5’-CTCGCCAAGCTAGCTTGG-3’. A positive colony containing the plasmid was selected, grown, induced and purified under the same conditions described for TcCat above and Figure S4. Bacteria transfected with pQE80 empty or pQE80TcCat were purified in parallel and aliquots of key steps in the purification were electrophoresed by SDS-PAGE and Coomasie blue stained (Fig. S5). The bands identified were cut from the gel (Fig. S5B, bands labeled A-D), trypsin digested and the proteins were identified by mass spectrometry to evaluate possible contamination with bacterial proteins (Table S1). Mass spectrometry identification was done at the Complex Carbohydrate Research Center (University of Georgia).

**References**

1. Sabirov RZ, Dutta AK, Okada Y (2001) Volume-dependent ATP-conductive large-conductance anion channel as a pathway for swelling-induced ATP release. J Gen Physiol 118: 251-266.

2. Bertl A, Ramos J, Ludwig J, Lichtenberg-Frate H, Reid J, et al. (2003) Characterization of potassium transport in wild-type and isogenic yeast strains carrying all combinations of trk1, trk2 and tok1 null mutations. Mol Microbiol 47: 767-780.

3. van Suylekom D, van Donselaar E, Blanchelot C, Do Ngoc LN, Humbel BM, et al. (2007) Degradation of the hexose transporter Hxt5p in *Saccharomyces cerevisiae*. Biol Cell 99:13-23 .

4. Molina ML, Encinar JA, Barrera FN, Fernandez-Ballester G, Riquelme G, et al. (2004) Influence of C-terminal protein domains and protein-lipid interactions on tetramerization and stability of the potassium channel KcsA. Biochemistry 43: 14924-14931.
